# Gaze allocation and evaluations of dynamic smiles following angry versus neutral expressions

**DOI:** 10.1371/journal.pone.0356283

**Published:** 2026-08-20

**Authors:** Shuang Wu, Yuki Shimizu, Xiyue Chen

**Affiliations:** 1 Department of Psychology, Graduate School of Letters, Arts and Sciences, Waseda University, Shinjuku-ku, Tokyo, Japan; 2 Faculty of Letters, Arts and Sciences, Waseda University, Shinjuku-ku, Tokyo, Japan; 3 Workplace Options China, Shanghai, China; New York University Abu Dhabi, UNITED ARAB EMIRATES

## Abstract

This study investigated how people perceive smiles that follow an angry versus a neutral expression by examining gaze patterns and subjective evaluations when presented with dynamic facial expressions. In an eye-tracking experiment, participants viewed morph movie stimuli in which facial expressions changed either from anger to a smile (anger-to-smile condition) or from a neutral expression to a smile (neutral-to-smile condition), while their gaze allocation to different facial parts (eyes, nose, mouth) was recorded. Then participants evaluated the sincerity of the final smiles and how happy and angry the poser appeared to feel. Results revealed that ratings of sincerity, happiness and anger for the final smiles did not differ between conditions, whereas gaze allocation did. Participants in the anger-to-smile condition fixated longer to the eyes; however, fixations to the eye regions did not reliably predict subjective ratings. Contrary to our predictions, longer fixation on the mouth were associated with higher ratings of sincerity and happiness regardless of conditions and with lower ratings of anger in the anger-to-smile condition. Time-course analyses for gaze distribution over time indicated that gaze shifted from the nose toward the eyes and mouth over time and exploratory analyses also showed that the temporal changes in the gaze distribution in later phases of morphing changes were associated with subjective ratings. However, these associations did not reliably differ by preceding emotional contexts. These findings suggest that, when viewing dynamic smiles embedded in a prior negative context, attention to the smiling mouth is linked to more positive evaluations, potentially reducing sensitivity to earlier negative emotional cues. In the present paradigm, the preceding facial expression did not produce detectable differences in explicit evaluations; whether such temporal information contributes to authenticity inferences remains to be tested directly. Future research should examine this question using explicit authenticity judgments and richer social-context cues that motivate masking.

## Introduction

Display rules of facial expressions dictate what emotional expressions are considered appropriate [1–3]. In interpersonal situations, there are many techniques manipulating and regulating emotional expressions, including facial expressions, to meet the display rules. Depending on the social contexts, inconsistencies could arise between how people express emotions externally and how they genuinely feel internally. One of the most used techniques regulating facial expressions that creates inconsistency is the masking of facial expressions, such as fake smiles that conceal negative emotions [3,4].

Analyses on how people express different types of smiles reveal that there are morphological differences between fake smiles and genuine smiles. Major morphological features of a smile including the contraction of the zygomaticus major muscle, which caused the upper lip raising and upper teeth exposing, and contraction of the inferior part of orbicularis oculi muscle, which caused the cheeks lifting, eye openings narrowing, and wrinkles around the eye region [2,5]. In contrast to the zygomaticus major muscle, the contraction of orbicularis oculi muscle is subjected less to the intentional, voluntary control and represents a more genuine smile [6,7]. Importantly, however, authenticity (“fake” vs. genuine) is rarely inferred from facial morphology alone. Observers also rely on contextual information, including what occurs immediately before the smile, to infer whether the smile reflects genuine positive affect. In the present study, we therefore treated a smile following anger as a potentially masking context.

Previous eye-tracking studies suggest that attention allocation to different facial parts was significantly associated with the subjective perception of smiles. For genuine smiles, the longer people fixate to the mouth, the happier they would rate the poser to be. However, when people are trying to discriminate fake smiles, longer fixation to the mouth might lead to biased perception. For example, Calvo, Fernández-Martín [8] emphasized the misleading effect of a salient smiling mouth on the perception of fake smiles, in which the participants rate the stimuli with a smiling mouth as happier even though the eyes are sad. On the other hand, movements around the eye region provide the key indicator of fake smiles and greater attention allocation to the eyes has been confirmed to improve the identification of fake smiles [9,10]. Del Líbano, Calvo [11] focused on the eye-mouth inconsistency and found that participants would rate the smiles with angry eyes, even with lower-intensity anger, as non-happy smiles than the smiles with neutral eyes. Combining with the results of the previous studies stating that muscular movements around the eye region occur more often when people are expressing genuine happiness instead of faking their happiness [4,5,12], and that people use mouth movements to cover up for a forced smile [13], there is a greater possibility to recognize a fake smile when we pay more attention to the eye region. Conversely, cues from the eye region are not perfectly diagnostic of smile authenticity. The so‑called Duchenne marker (orbicularis oculi activation) can be produced deliberately in posed or socially motivated smiles, and its presence shows limited specificity to felt enjoyment, partly because it covaries with overall smile intensity [14–16]. Therefore, defining “fake smiles” primarily via extreme eye–mouth incongruence (e.g., a smiling mouth with non‑smiling eyes) may oversimplify real-world cases, in which the eyes may also “smile” despite non‑genuine intent. This motivated the present study to examine how observers integrate dynamic information and preceding emotional context when interpreting smiles.

Moreover, the temporal dynamics of how gaze shifts among different facial parts were linked to the perceived emotions of the smiles. For example, Calvo, Gutiérrez-García [17] revealed that early attention is also important when discriminating fake smiles from genuine smiles. When the first fixation is directed to the mouth, participants are more likely to judge the smiles as genuine.

Yet previous work provides only limited evidence on how temporal context—that is, what facial expressions precede a target expression—shapes observers’ perception of that expression. This issue is particularly relevant to judgments of smile authenticity, because a smile can function as a social mask for preceding negative affect (i.e., a non-genuine or “fake” smile), and thus the immediately preceding expression may provide a critical cue for interpreting the smile. Studies by Fang and colleagues had systematically manipulated sequences of emotional expressions using static and dynamic faces and demonstrated effects of current versus past emotions and cultural background on emotion and person perception [18–21]. Nevertheless, their paradigms typically involved transitions between different basic emotions (e.g., neutral-to-happy, happy-to-sad) and focused on overall evaluations of the changing emotional states, rather than on smiles that may be interpreted as masking prior negative affect. From another perspective, Iwasaki and Noguchi [13] employed stimuli with the mouth and eye regions changing in separate timings and revealed that movements in the mouth region can bias the recognition of micro-expressions in the eye region. However, these studies did not examine gaze allocation. Thus, it remains unclear whether, and at which time points, fixations to different facial features during changing expressions influence the perceived authenticity of smiles that follow a negative versus neutral expression. Ambadar, Cohn [22] examined the dynamic characteristics of different smiles (onset and offset velocity, amplitude in head movement, duration of smiles and others) and how they affect the perceived meaning of the smiles. They argued that, compared with static faces, dynamic faces provide detailed motion cues that enhance the detection of fake smiles. Taken together, these studies highlight the importance of dynamic information but leave the open question of how temporal context and gaze behavior jointly shape the perception of smiles that may conceal prior negative emotions. Therefore, it remains an important task in filling the gaps in the literature to use dynamic stimuli that simulate changes from negative or neutral expressions to smiles and examine the time course of attention allocation.

Building on these insights, the current study employed dynamic stimuli in which facial expressions change either from anger to a smile or from a neutral expression to a smile to investigate the relationship between gaze dynamics and subjective evaluations. We aimed to elucidate the psychological processes involved in the perception of smiles that occur in different temporal contexts. A transition from anger to a smile can be interpreted in multiple ways. It may signal deliberate masking of prior negative affect, but it may also reflect emotion regulation and recovery, interpersonal appeasement, or a shift in social intent. Because our paradigm manipulated only the immediately preceding facial expression and did not provide explicit situational cues about motive, therefore, our key question was not whether observers can conclusively label smiles as ‘fake’ or ‘genuine,’ but how temporal context shapes gaze allocation and the evaluative weighting of facial cues during dynamic perception.

We first hypothesized that temporal context (i.e., the expression preceding the smile) would influence cognitive judgments of the final smiles. Specifically, a smile following an angry expression (anger-to-smile condition) may be perceived as a “masking” (potentially fake) smile that conceals residual negative affect, whereas a smile following a neutral expression (neutral-to-smile condition) is less likely to imply such masking effect. Accordingly, if observers interpret anger-to-smile sequences as indicating residual negative affect or masking, they should rate the final smiles as less sincere, less happy and angrier than smiles changing from neutral expressions.

Second, we hypothesized that the visual attention toward the final smiles would also differ based on preceding facial expressions. We predicted that participants would fixate longer to the eyes of the posers when their expressions change from anger to smiles compared to when their expressions change from neutral to smiles.

The third hypothesis concerned the association between visual attention to the eyes and subjective evaluations of the final smiles. We predicted that longer fixation to the eyes would associate with less sincerity, less happiness and greater anger evaluation in the anger-to-smile condition compared to the neutral-to-smile condition. This prediction was made based on previous studies revealing that people tend to look longer at the eyes and use the diagnostic cues from the eye region especially when interpreting negative facial expressions, such as angry or fearful expressions [23–25]. Considering the importance of the eye region in the discrimination of fake smiles, we predicted that greater attention to the eyes would correlate with more negative evaluations of smiles that follow an angry expression.

The fourth hypothesis focused on the time course of attention. We first predicted that the attention to the eyes would increase over time in the anger-to-smile condition compared to in the neutral-to-smile condition. We also predicted that greater attention to the eyes during the early phase—when information about the preceding expression is still available—contribute more strongly to predicting the outcomes of the subjective evaluations. Although direct evidence for this hypothesis is limited, prior eye‑tracking research suggests that early fixations are particularly diagnostic in judging smile genuineness [17]. To make early‑phase information diagnostic in the present paradigm, we designed the stimuli in the present study such that the initial facial expressions differed between conditions (angry-to-smile condition and neutral-to-smile condition). Accordingly, we predicted that participants who allocate greater attention to the early phases of the changing facial expression—especially to the eyes in the anger‑to‑smile condition—would evaluate the final smiles as less positive (i.e., lower sincerity and happiness and higher anger ratings), indicating that their judgments are more strongly influenced by the temporal context. As previous studies also emphasize the importance of attention to the mouth region when evaluating genuine smiles, we would also conduct exploratory analyses on the relationship between subjective evaluations and time course of visual attention to the mouth.

Overall, the aim of this work was to test whether the expression preceding a smile (anger vs. neutral) shapes gaze allocation and the time‑resolved gaze–evaluation relationship during dynamic smile perception. The results reported below address this aim by showing context‑related shifts in gaze allocation and time‑dependent links between mouth attention and perceived anger, even though overall evaluation differences between conditions were not detected. In doing so, this study highlights that the integration and processing of contextual information in emotional perception might differ in terms of gaze behavior and evaluations.

## Materials and methods

### Participants

We analyzed data from 52 Japanese undergraduates (15 males, 37 females, *M*_*age*_ = 20.19, *SD*_*age*_ = 1.17) whose native language is Japanese and who reported normal or corrected-to-normal vision. The period of main data collection was from July 3rd, 2023, to January 17th, 2025. Participants were randomly assigned to the anger-to-smile condition (n = 27) or neutral-to-smile condition (n = 25). One participant assigned to the neutral-to-smile condition was excluded from final analyses due to insufficient looking time (total visit durations to the monitor screen less than 70% of the stimulus presentation duration) and was not included in the sample size above. In addition, we excluded 9.13% (38 out of 416) of the trials due to insufficient looking time. Based on a priori power analysis using G*Power [26] for the between-group comparisons (α = .05), a minimum sample size of 44 participants was needed to have sufficient power (>.80) to detect medium effects. Therefore, our final sample size can be considered sufficient for the planned basic between-condition comparison. Participants were recruited from introductory psychology courses and received course credits upon completion of the experiment. We first completed the initial briefing and obtained written informed consent from the participants before proceeding to the experimental tasks. All procedures for this study were approved by the Ethics Committees of Waseda University (No.2023−027).

### Materials

**Fig 1:**
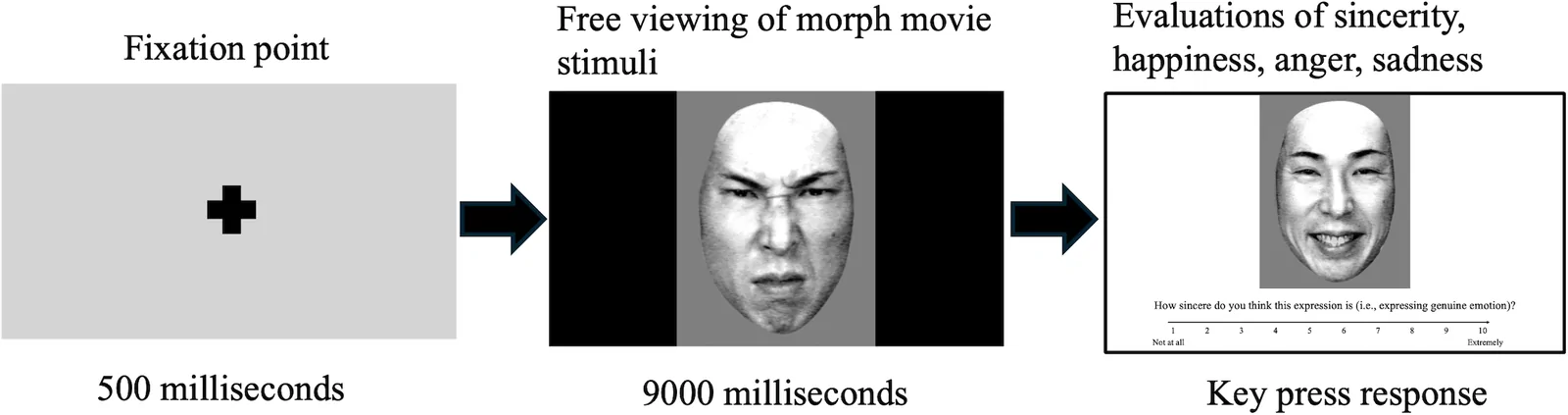
Flow of procedures for one experimental trial. For each one of the trials, the participants were first asked to fixate at a fixation cross, which was presented for 500 milliseconds, every time it appeared in the middle of the screen. Then, participants were asked to freely view the 9000-millisecond movie stimuli, which automatically started to play. Afterwards, participants were asked to press the number keys (0-9) to evaluate in a 10-point Likert-type scale about the ending smiles in terms of how sincere they think the final smiles were and the intensities of happiness, anger and sadness that they think the posers were feeling right now. The number key “0” means “10 (extremely)” in the evaluations, and the participants were briefed about it before the tasks started. Republished from ATR Facial Expression Image Database DB99 under a CC BY license, with permission from ATR-Promotions Inc., original copyright (2006).

**Fig 2:**
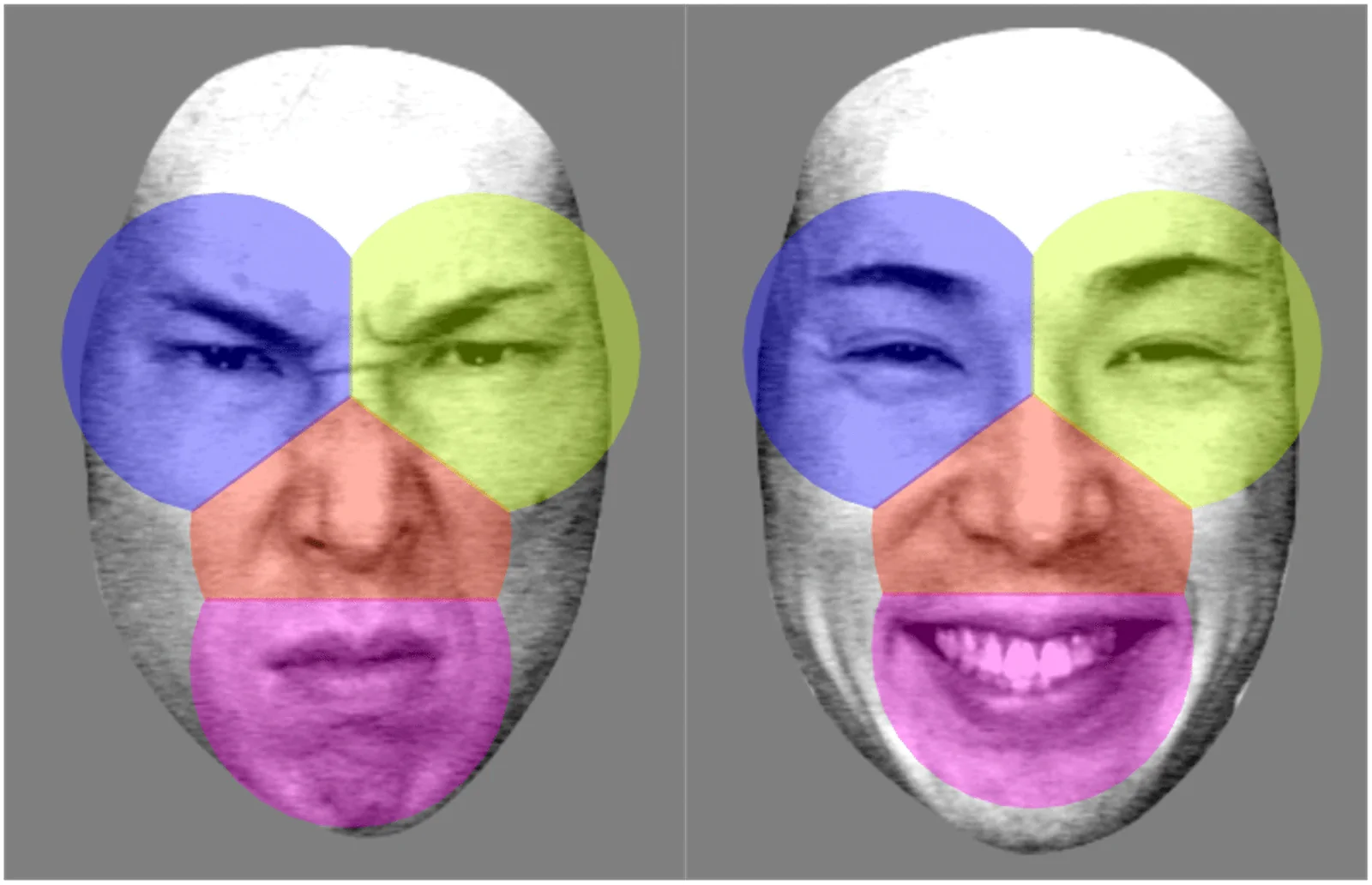
Examples of Areas of Interest (AOIs) for the Angry and Happy Expressions of a Japanese Male Poser. AOI centers (left eye, right eye, nose and mouth) were first determined, and the average distance between each of the two adjacent AOI centers was computed as AOI span. The radii of the AOIs were determined to be 75% of the AOI span. AOIs were manually drawn using AOI tools in Tobii Pro Lab with the positions fixed based on corresponding AOI centers and the sizes determined by the radii of the AOIs. For adjacent AOIs that converged, the AOIs were divided along the perpendicular bisectors of the line segments connecting the two corresponding AOI centers. Dynamic AOIs were adjusted by defining two key frames, the starting and ending frames of the change, and creating the AOIs for each of the two key frames. The feature of dynamic AOI in Tobii Pro Lab automatically computed the intermediate changes between the two key frames. Republished from ATR Facial Expression Image Database DB99 under a CC BY license, with permission from ATR-Promotions Inc., original copyright (2006).

We selected 32 still images of neutral, happy, angry, and sad expressions of 4 posers (2 Japanese males and 2 Japanese females) from ATR Facial Expression Image Database (ATR-Promotions) and of 4 posers (2 Chinese males and 2 Chinese females) from intensity-classified Chinese facial emotion image database [27]. Previous studies examining facial expression perception of East-Asian participants either use East-Asian stimuli that do not specify the nationality or manipulate the stimuli to consist of only lines depicting basic facial features [10,13,28]. Therefore, to increase the variety of the stimuli, we used stimuli of both Japanese and Chinese posers so that our results would be more suited to compare with those of previous studies. For each image, we removed non-facial features, such as hairs and clothing, and converted the colorful images into black and white ones of 350 × 450 pixels (width × height) using Photoshop. These still images were validated through an online pilot study with twenty-three Japanese participants (see S1 File for detailed descriptions on the pilot study). Among the chosen facial expression images, two images from one Japanese poser were used in Figs 1 and 2 in the following sections. ATR Promotions, as the owner of the images, has given written permission to publish the two images under CC-BY license. Using FantaMorph ver.5.4.8 (http://www.fantamorph.com), we created 24 morph movie stimuli (9-second-long with 135 frames), with 8 (2 posers × 2 stimulus’ cultures × 2 stimulus’ genders) for the anger-to-smile condition, 8 (same as above) for the neutral-to-smile condition, and 8 (same as above) for the smile-to-sadness filler tasks. The final smile was identical across different conditions for each poser. These filler trials were included to prevent participants from anticipating that every trial would end in a smile and potentially causing biases in their evaluations, thereby encouraging more naturalistic viewing of the entire sequence. The results for the filler tasks were not included in the final analysis. Each morph movie consisted of 90-frame (6 seconds) gradual changes of facial expressions and 45-frame (3 seconds) presentation of the still image of the ending expression. We determined the localizers for the key features of the faces, and the software computed the linear and continuous changes of the corresponding localizers from the starting to ending frames at the speed of 15 frames per second. The participants were randomly assigned to either the anger-to-smile condition or the neutral-to-smile condition. They were presented with the stimuli of each corresponding condition as well as the stimuli of filler tasks, in a randomized order.

### Procedure

Each participant was tested individually in-person in a lab setting and was seated in front of a monitor (Dell 23.8-inch; pixel resolution of 1920 × 1080). To maintain a viewing distance of 60 cm, participants were asked to adjust the seating and fix their head position with a chin rest. The eye-tracking data was collected with Tobii Pro X3-120 (Tobii Technology, Sweden) positioned at the bottom of the monitor at a sampling rate of 120 Hz and the experiments were conducted using Tobii Pro Lab. When exporting eye-tracking data, the gaze filter I-VT (Fixation) was selected and the minimum requirement for fixations is a fixation duration of no less than 60 milliseconds. The participants first completed a nine-point calibration with re-calibration or re-validation if necessary. During the experiment, the movie stimuli subtended approximately 9.15° × 11.75° in visual angle (width × height).

The participants completed one practice trial, followed by eight target trials for the assigned condition (4 Japanese and 4 Chinese posers; 4 female and 4 male) and eight filler trials (smile-to-sadness). Only the eight target trials were included in the analyses. Each trial was comprised of a free viewing task and an evaluation task. During the free viewing task, the participants were asked to freely view the entire 9-second morph movie following a 0.5-second fixation cross presented in the middle of the screen. The order of the morph movie was randomized for each participant. Then, in the evaluation task, the participants were asked to evaluate the sincerity, happiness, anger, and sadness of the ending facial expression presented in the morph movie and respond by key pressing in a 10-point Likert scale (1: not at all – 10: extremely). The questions being presented were “How sincere do you think this expression is (i.e., expressing genuine emotion)?” and “How happy/angry/sad do you think this individual is feeling right now?”. Fig 1 shows the flow of procedures of one experimental trial. The question regarding sadness was designed only for the filler tasks and the ratings of sadness were not included in the final analyses.

### Data analysis

#### Areas of interest (AOIs).

We used a noise-robust method called the limited-radius Voronoi tessellation method (LRVT method; 29) to draw areas of interests (AOIs) for left eye, right eye, nose and mouth for each stimulus (see Fig 2 for examples of AOIs). The size of the AOI was determined to be 75% of the AOI span, which was the average distance between adjacent AOI centers. This coefficient was determined based on Hessels et al.’s [29] study revealing that, for sparse stimuli such as faces, the amount of eye-tracking data included in AOIs stabilizes when the size reaches 75% of the AOI span. The AOIs were also adjusted to the dynamic stimuli by using the feature of dynamic AOIs in Tobii Pro Lab. For the final analyses, the eye-tracking data for the left and right eyes was combined as “eyes” region. The sum of total fixation duration to the AOIs (eyes, nose and mouth) is on average 17 milliseconds less than the total fixation duration to the screen. The eye-tracking metrics of calibration and validation accuracy and precision are reported in S1 Table.

#### Statistical analysis.

All statistical analyses were conducted using R (version 4.5.0).

For the first hypothesis regarding the evaluation of final smiles, we fitted linear mixed-effect models (LMMs) for each of the evaluations (sincerity, happiness and anger) with the main effects of condition and stimulus’ culture and with the main effects and interaction of participant’s gender and stimulus’ gender as fixed effects and random intercepts of participant ID (1 | Participant) and stimulus media (1 | Media). According to the results of the residual diagnostics for all three ratings, we observed ceiling effects for the ratings of sincerity (*p* = .003) and happiness (*p* = .017) as well as a floor effect for the rating of anger (*p* < .001). To account for the skewness of the data, we also fitted the ordinal mixed models for each of the ratings, with condition and stimulus’ culture as predictors and with the same random effects as in LMMs.

For the second hypothesis examining how the gaze allocation varied across different AOIs (eyes, nose, mouth) and between conditions, we fitted a LMM to the total fixation duration. Fixed effects included the main effects and interaction of condition and AOI as well as the main effect of stimulus’ culture. Random effects included random intercepts for participant ID (1 | Participant) and stimulus media (1 | Media).

For the third hypothesis concerning the relationship between fixation durations and evaluation scores, we fitted LMMs for each of the ratings with total fixation duration to each AOI, condition and their interactions as well as stimulus’ culture and total fixation duration to the face as fixed effects, and with random intercepts for participant ID (1 | Participant) and stimulus media (1 | Media). Only for the rating of anger, the stimulus media had extremely small variance and was dropped from the final model. The results and goodness of fit for the simplified model (AIC = 1148.60) did not differ much from the original more complex model (AIC = 1150.60). Therefore, the results for the final simplified model were reported for the rating of anger.

For the first half of the fourth hypothesis, to examine the time course of changes in the visual attention, we divided the 9-second movie stimuli into nine consecutive time bins of 1000 milliseconds. The first six time bins [1–6] corresponded to the morphing phase, and the last 3 bins [7–9] corresponded to the static final expression. A LMM was then fitted to the fixation duration toward each AOI in each time bin. Fixed effects included main effects and interactions of condition, time bin, and AOI, main effect of stimulus’ culture, and random effects were consisted of a participant ID random slope for time bin (1 + Time bin || Participant) and a random intercept for stimulus media (1 | Media).

For the second half of the fourth hypothesis examining the relationship between ratings of the final smiles and the time course of fixation durations to the eyes, we first divided the movie stimuli into three consecutive time bins of 3000 milliseconds. The first time bin included frames of early morphing phase, the second time bin included frames of late morphing changes, and the third time bin included the static frames of the final expressions. We then fitted LMMs for each of the ratings, with the main effects and interactions of binned fixation duration to the eyes in each time bin and condition, the main effects of fixation duration to the face in each time bin as fixed effects, and with random intercepts for participant (1 | Participant) and stimulus media (1 | Media). In addition, as an exploratory analysis, to examine the relationship between subjective evaluations and time course of visual attention to the mouth, we fitted the LMMs for each rating using binned fixation duration to the mouth, condition, and their interactions as fixed effects.

For the models above, to account for the differences brought by the stimulus’ culture, we included it as one of the fixed effects. However, since the focus of the current study is not on the cultural differences of the perception of fake smiles, we only examined the main effect of stimulus’ culture but not its interaction effects with other factors. Also, the total fixation duration and binned fixation duration were normalized via log-transformation using log(x + 1) function and standardized into z-scores. Condition, AOI, stimulus’ culture, participant ID and stimulus media were treated as factors. For models that included binned fixation metrics, time bin was centered as a continuous numeric variable. Categorical predictors were sum-coded (intercept = grand mean). For significant interactions, holm-adjusted post-hoc analyses or simple slope analyses were performed using *emmeans* package.

### Inclusivity in global research

Additional information regarding the ethical, cultural, and scientific considerations specific to inclusivity in global research is included in the Supporting Information (S1 Checklist).

## Results

### Descriptive statistics

**Fig 3:**
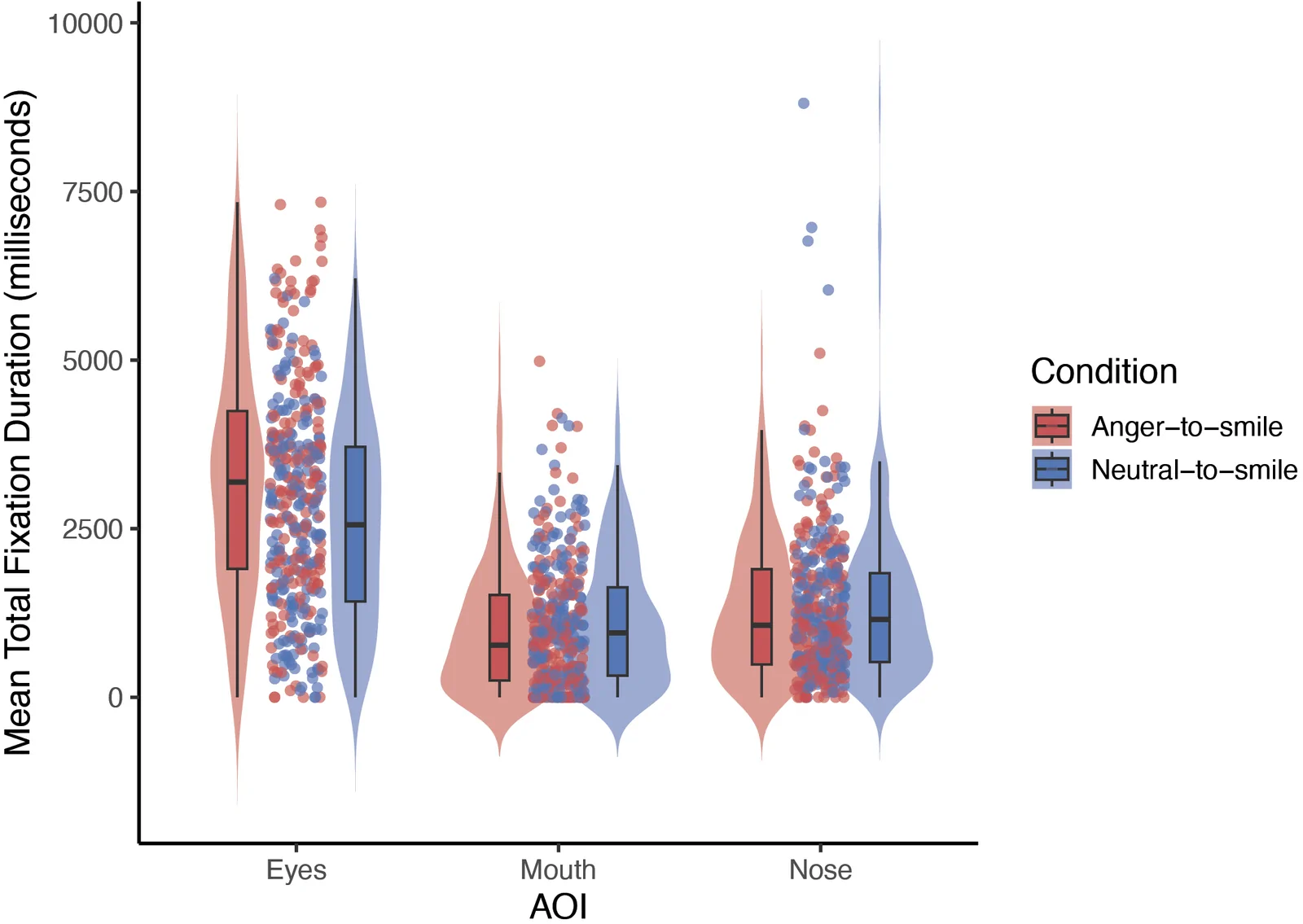
Mean Total Fixation Duration to Different AOIs (Eyes, Nose, Mouth) in Neutral-to-Smile Condition and Anger-to-Smile Condition. Red represents anger-to-smile condition and blue represents neutral-to-smile condition. Dots represent data points of each trial’s total fixation durations.

The means (*SD*s) for the evaluations of sincerity, happiness and anger in each condition are shown in Table 1 and S1 Fig. The mean (*SDs*) total fixation durations to each AOI are shown in Table 1 and Fig 3.

**Table 1:** Means and Standard Deviations of Evaluation of the Smiles and Total Fixation Duration (milliseconds) to Each AOI.

	Neutral-to-smile condition		Anger-to-smile condition	
	Mean	SD	Mean	SD
Sincerity	6.74	2.24	6.98	2.02
Happiness	7.22	2.22	6.94	2.02
Anger	1.53	0.95	1.81	1.43
Fixation Duration (Eyes)	2613.89	1473.29	3219.36	1701.17
Fixation Duration (Nose)	1381.95	1235.19	1286.13	997.32
Fixation Duration (Mouth)	1090.36	927.65	973.40	932.77

*SD* represents standard deviation.

### Evaluation of the final smiles

We performed separate LMM analyses for each of the evaluations (sincerity, happiness and anger) to examine whether there were any differences between conditions. Condition (sincerity: *b* = −.12, SE = .24, 95% CI [−.61,  .36], *p* = .607; happiness: *b* = .16, SE = .27, 95% CI [−.38,  .70], *p* = .553; anger: *b* = −.12, SE = .11, 95% CI [−.35,  .11], *p* = .292) and stimulus’ culture, participant’s and stimulus’ gender were not significant for all models. The detailed results are reported in S2 Table. The results of ordinal mixed models (see S3 Table) for all three ratings were also consistent with those from LMMs.

### Total fixation duration

We performed an LMM analysis for the total fixation duration to examine the effects of AOI and condition on the overall attention allocation when viewing the movie stimuli. Fixation duration differed across facial regions: the eyes deviation from the grand mean was positive (b = .48, SE = .04, 95% CI [.41,  .55], *p* < .001), whereas the nose deviation was near zero (b = −0.01, SE = 0.04, 95% CI [−.08,  .06], p = .812), implying the mouth region received the least fixation overall. Consistent with the descriptive statistics (Table 1; Fig 3), fixation duration was highest for the eyes and lowest for the mouth. The interaction of AOI and condition was also significant (b = −.09, SE = .04, 95% CI [−.16, −.02], *p* = .012), indicating that the pattern of fixation across AOIs differed between conditions; descriptively, participants in the anger-to-smile condition showed relatively greater fixation to the eyes than participants in the neutral-to-smile condition (Fig 3). The detailed results are shown in S4 Table.

### Time course of visual attention

**Fig 4:**
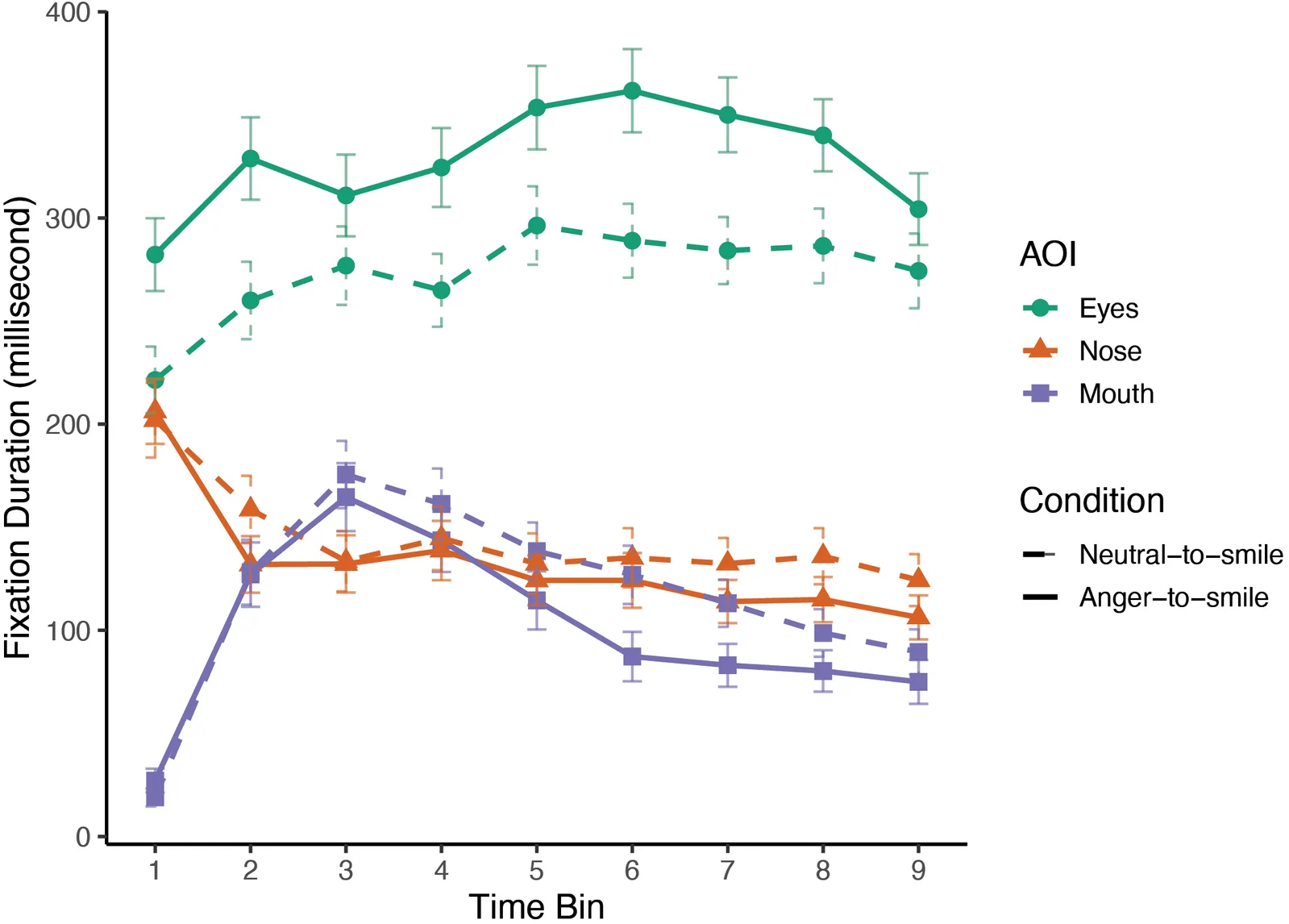
Fixation Duration to Different AOIs (Eyes, Nose, Mouth) Over Nine Time-Bins. Error bars represent standard errors. Dashed line represents neutral-to-smile condition and solid line represents anger-to-smile condition.

Fig 4 shows the time course of changes in the fixation duration to different AOIs in two conditions. A LMM revealed a robust main effect of AOI, indicating that the eyes AOI showed a positive deviation from the grand mean (b = 1.37, SE = .03, 95% CI [1.30, 1.43], *p* < .001), whereas the nose AOI showed a negative deviation (b = −.35, SE = .03, 95% CI [−.42, −.28], *p* < .001), implying that fixation was lowest for the mouth overall (Fig 4). The AOI × Condition and Time bin × AOI interactions were also significant. For the AOI × Condition interaction, compared to participants in the neutral-to-smile condition, participants in the anger-to-smile condition allocated relatively more gaze to the eyes (b = −.20, SE = .03, 95% CI [−.27, −.14], *p* < .001). For the Time bin × AOI interaction, gaze shifted over time for the nose (b *=* −.06, SE = .01, 95% CI [−.09, −.04], *p* < .001) and increased for the eyes (b = .04, SE = .01, 95% CI [.01,  .06], *p* = .003), which implies a corresponding relative increase for the mouth. However, the overall three-way interaction among condition, time bin, and AOI was not significant. Detailed results of the LMM are shown in S5 Table.

### Relationship between evaluations and fixation duration

We performed separate LMM analyses for each evaluation (sincerity, happiness, and anger) to examine how total fixation durations toward each AOI in each condition were associated with the perception of the final smiles. Detailed results are shown in S6 Table.

There was a significant main effect of fixation duration to the mouth for the evaluations of sincerity (*b* = .28, SE = .12, 95% CI [.04,  .51], *p* = .023), happiness (*b* = .27, SE = .11, [.04,  .49], *p* = .019) and anger (*b* = −.22, SE = .07, 95% CI [−.35, −.09], *p* = .001), revealing that participants who fixated longer to the mouth rated the final smiles as more sincere, happier and less angry. Also, for the evaluations of anger, there was a significant main effect of fixation duration to the nose (*b* = −.16, SE = .08, 95% CI [−.32,  .00], *p* = .050), indicating that participants who fixated longer to the nose also rated the smiles as less angry. The total fixation to the face was significant as well (*b* = .21, SE = .10, 95% CI [.02,  .40], *p* = .030), indicating that participants who overall fixated longer during the stimulus presentation rated the smiles as angrier. There were also significant interactions of fixation duration to the nose and Condition (b = −.15, SE = .07, 95% CI [−.30,  .00], *p* = .044), and of fixation duration to the mouth and Condition (*b* = .13, SE = .06, 95% CI [.00,  .26], *p* = .048). Simple slopes revealed that participants in the anger-to-smile condition who fixated longer to the mouth rated the final smiles as less angry (estimate = −.35, SE = .08, 95% CI [−.58, −.19], *p* < .001), whereas fixation duration to the nose in the neutral-to-smile condition was associated with less perceived anger (estimate = −.31, SE = .14, 95% CI [−.58, −.04], p = .048).

### Effects of time course of visual attention on evaluations

To examine time-varying associations between gaze allocation and subjective ratings, we first performed separate LMM analyses for each of the evaluations (sincerity, happiness, and anger), with the main effects and interactions of condition and fixation duration to the eyes in early morphing phase (bin 1), late morphing phase (bin 2) and final static phase (bin 3), including stimulus’ culture and fixation duration to the face in each time bin as covariates. The detailed results are in S7 Tables. We did not observe significant main effects or interactions with condition. Therefore, the marginally significant main effects of binned fixation duration to the eyes in bin 2 for the ratings of sincerity (*b* = −.28, SE = .15, 95% CI [−.58,  .03], *p* = .073) and happiness (*b* = −.28, SE = .14, 95% CI [−.56,  .00], *p* = .052) could only suggest potential negative associations between fixation to the eyes in late morphing phase and ratings of sincerity and happiness,

In the additional exploratory analyses, we explored the relationship between time course of gaze allocation toward the mouth region and subjective evaluation. We performed separate LMM analyses for each rating with time-binned fixation duration to the mouth and the interaction with condition included in the fixed effects. The detailed results are reported in S8 Table. The significant results observed were as following.

For the evaluations of sincerity and happiness, the main effect of binned fixation duration to the mouth in bin 2 was significant (sincerity: *b* = .34, SE = .13, 95% CI [.09,  .59], *p* = .007; happiness: *b* = .30, SE = .12, 95% CI [.07,  .53], *p* = .012), indicating that participants who fixated to the mouth longer during the late morphing phase of the stimuli would rate the final smiles as more sincere and happier. The interaction of binned fixation duration to the mouth in bin 2 and condition was also significant for the evaluations of sincerity (*b* = −.34, SE = 13, 95% CI [−.59, −.09], *p* = .008) and happiness (*b* = −.26, SE = .12, 95% CI [−.50, −.03], *p* = .030). Simple slope analyses revealed that the slopes were significant only in the neutral-to-smile condition for both the evaluations of sincerity (slope = .58, SE = .20, 95% CI [.19,  .97], *p* = .007) and happiness (slope = .51, SE = .18 95% CI [.15,  .87], *p* = .011), indicating that the positive association between fixation to the mouth in late morphing phase and subjective ratings was strong in neutral-to-smile condition but not in anger-to-smile condition. For the evaluations of anger, the main effects of binned fixation duration to the mouth and face in bin 3 were significant (mouth: *b* = −.21, SE = .06, 95% CI [−.33, −.08], *p* = .001; face: *b* = .17, SE = .08, 95% CI [.01,  .33], *p* = .037). The results indicated that during the presentation of static final smiles, participants who fixated longer to the mouth would rate the smiles as less angry. Across all models (S7–S8 Tables), condition did not reliably moderate the fixation–evaluation slopes or their change across time bins, suggesting that the time-varying fixation–evaluation associations were broadly similar between the anger-to-smile and neutral-to-smile conditions.

## Discussion

This study examined how visual attention and subjective judgement of the dynamic simulations of smiles changing from angry or neutral expressions differ and how attention allocation to different facial parts was associated with the subjective judgement of the final smiles. We also explored how the relationship between attention allocation and subjective ratings change over time.

### Subjective evaluations of smiles following anger versus neutral expressions

Regarding the evaluations of the final smiles, our hypothesis was not supported as there were no significant differences in the evaluations between conditions. One possibility for the null results for perceived sincerity and happiness is that the initial neutral expressions in the neutral-to-smile condition may not have been experienced as truly neutral by Japanese observers. Cross‑cultural evidence shows that Japanese participants tend to attribute relatively negative or intense emotions to faces that Western participants perceive as neutral: for example, they rate neutral faces with direct gaze as angrier, less approachable, and less pleasant than Finnish observers do [30], and they judge neutral faces as overall more emotional, presumably because neutral expressions are interpreted as masking suppressed emotion [31]. If our participants similarly perceived the initial neutral faces as slightly negative or emotionally loaded, the contrast between the anger‑to‑smile and neutral‑to‑smile conditions would have been reduced, thereby attenuating condition differences in the evaluations of the final smiles. Another possible explanation is that, as Fang, van Kleef [32] have suggested, there is a strong and consistent recency effect when judging changing emotional expressions. Therefore, in the anger-to-smile condition, the participants might focus too much on the high-intensity smiles at the end and may not fully take the initial angry expressions into consideration when making their evaluations, especially for the positive emotions.

### Visual attention to smiles following anger versus neutral expressions

The second hypothesis regarding differences in the visual attention to smiles changing from angry or neutral expressions was supported. We observed greater attention allocation to the eyes of the stimuli consistent across conditions as well as significant differences between conditions in the differential attention to the eyes and mouth. Specifically, the participants fixated relatively longer to the eyes when presented with smiles changing from angry expressions compared to smiles changing from neutral expressions. The greater attention to the eyes when interpreting facial expression was in line with the results of previous studies [33–35] stating that Japanese participants tend to focus more on the cues from the eyes when interpreting facial expressions. The differences in the attention allocation between conditions elucidated an important influence of the temporal contexts preceding the smiles on the attention allocation to different facial parts.

### Relationship between visual attention and subjective evaluations of smiles

The third hypothesis predicting the relationship between visual attention to the eyes and the ratings of the final smiles was not supported. LMM results did not show any associations between fixation duration to the eyes and the ratings of sincerity, happiness, and anger. In contrary to what we predicted, we found positive associations between fixation duration to the mouth and perceived sincerity and happiness as well as a negative association between fixation duration to the mouth and perceived anger observed only in the anger-to-smile condition. We also found a negative association between fixation duration to the nose and perceived anger in the neutral-to-smile condition. The nose region affords a holistic overview of the face, and in the absence of a preceding angry context, greater holistic sampling may have reinforced perception of the final expression as a coherent genuine smile, thereby lowering attributions of anger.

The results were not in agreement with the previous studies [9,10] that concluded greater attention to the eyes improves the identification of fake smiles. One possibility is that, as multiple previous research have also suggested, greater attention to the mouth could bias and increase the perceived genuineness of the smiles [8,17,28], partially explaining why we did not observe any difference in the evaluations of sincerity and happiness between conditions. Moreover, the result that greater attention to the mouth was associated with lower perceived anger in the anger-to-smile condition is in line with the results from Iwasaki and Noguchi [13], which suggest that people tend to be biased by the smiling mouth when they recognize the changing mouth and eyes as one unified object rather than disconnected facial parts providing different emotional cues. The attention bias to a smiling mouth may be robust as it disrupts the recognition of the changes in the eye region regardless of the emotional valence. In the present study, despite the preceding expressions being negative or neutral, the unified changes in the facial features might enhance positive attention bias toward the smiling mouth and reduce weighting of the preceding negative contexts on the judgement of the final smiles.

### Time course of visual attention and its links to evaluations

Regarding the first half of the fourth hypothesis stating that the attention to the eyes in the anger-to-smile condition would increase over time was not supported. In line with the LMM results for total fixation duration, we found that participants allocated greater attention to the eyes compared to the mouth in anger-to-smile condition even when we divided the attention into short intervals. We have also observed significant differences in how attention to different facial parts shifted over time, with gaze shifting from nose to eyes and mouth over time. However, we did not observe significant three-way interactions with condition. Nonetheless, the differences in the time course of how attention shifted offer a different perspective to investigate how attentional bias to different facial parts changes over time. This approach allows us to focus more on when and to which facial parts the attention shifts during the entire processing of smiles, providing more insights on the mechanism compared to studies that have only focus on the initial attention (i.e., the first fixation) or the total dwell time [9,17,25].

The second half of the fourth hypothesis regarding the association between the ratings of the final smiles and the attention to the eyes in the early phase was not supported, as we only observed marginally significant main effects for the binned fixation duration to the eyes and did not observe any interactions with condition. In the exploratory LMM models for the binned fixation duration to the mouth, we instead observed that positive associations for the perceived sincerity and happiness, especially in the neutral-to-smile condition and an overall negative association for the perceived anger. These associations were observed in later morphing phases when the smiles were more prominent, or the presentation of static final smiles. The results again agreed with previous studies suggesting the strong influence of attention to the mouth region on the perceived genuineness of smiles [8,17,28], strengthening our findings regarding hypothesis 3. More importantly, the results were in contrary to the finding of Calvo, Gutiérrez-García [17] that early fixations hold more diagnostic value in discriminating fake smile from genuine smile. In addition to the possible recency effect, potentially affecting the ratings of the final smiles, and Japanese people’s tendency to allocate more attention to the eyes, leading to less variance in the fixation duration to the eyes across different time bins, the model of the diagnostic early fixation might not be sufficient to explain the perception of smiles, especially when preceded with conflicting temporal contexts. Overall, these results suggest that temporal context may change how attention is deployed across facial regions over time, whereas the gaze–evaluation links appear comparatively stable across contexts.

Moreover, the discrepancies between in how gaze allocation and evaluations differ by preceding emotional expressions suggest that the spontaneous eye movements and subjective social-emotional evaluations reflect different processes. As in real-life, how we perceive dynamically conflicting and consistent facial expressions might require us to pay more attention to the most salient part or the more rapidly changing facial features. Therefore, the visual attention may be shaped more in a bottom-up and less consciously controlled way. On the other hand, making social-affective inferences about smile sincerity or the current emotional state might require one to integrate surrounding information in a more top-down way, influencing by personal beliefs, cultural norms or social-emotional situations. We are also interested in examining how people perceive mixed emotions from the changes in facial expressions, as cultural experiences and norms regarding the socially appropriate emotional expressions are likely to shape how people perceive and recognize emotions.

### Implications for the perception of smiles in potentially masking contexts

The present findings imply that when a smile is preceded by a potentially negative context, observers allocate relatively more attention to the eye region—consistent with the idea that viewers sample eye-region cues when authenticity or underlying affect is uncertain. This eye-focused approach is supported by studies indicating that attention to the eyes enhances the ability to distinguish between genuine and fake smiles [9,10]. Additionally, evidence suggests that activity around the eyes is less susceptible to voluntary control and is associated with enjoyment smiles [6,7]. However, eye-region cues are not perfectly specific to felt enjoyment: orbicularis oculi activation can be produced deliberately [14–16] and tends to covary with overall smile intensity, which may limit its utility as a standalone authenticity marker in the absence of richer situational information. This may help explain why increased eye monitoring in the anger-to-smile condition did not translate into systematically lower sincerity or happiness ratings in the present study. At the same time, the present pattern highlights the potential dominance of mouth cues in evaluation: a salient smiling mouth can bias the interpretation of eye-region information [8], and mouth movements may even conceal subtle diagnostic signals from the eyes [13]. Thus, increased eye monitoring in a potentially masking context does not necessarily translate into stronger or more negative judgments; instead, observers may sample the eyes for diagnostic cues while still relying heavily on mouth-related positivity when forming final evaluations.

The second implication concerns the temporal aspects of the relationship between attention allocation and explicit evaluations. Based on the results of the exploratory analyses, increased attention to the smiling mouth, when it became salient during the later phase of changing facial expressions, was linked strongly to higher perceived sincerity and happiness as well as lower perceived anger. This result is consistent with the positive attention bias of the smiling mouth, as suggested by previous studies, on smile perception, in terms of reducing the weighting of incongruent contexts [8,13,28]. It also agrees with the evidence that observers integrate information across changing facial expressions, often showing strong weighting of the current (or most recent) expression during person and emotion perception [20,32].

Finally, these results underscore the value of combining dynamic, context-manipulated stimuli with time-resolved eye-tracking to study masking-related interpretations of smiles. Prior work has emphasized early fixations and eye-region cues in judging smile genuineness [17], and dynamic information has been shown to provide additional timing and motion cues for interpreting different smile meanings [22]. Extending these approaches to temporally conflicting sequences (e.g., anger-to-smile) may help refine models of how gaze, facial-feature cues, and temporal context jointly contribute to judgments of smiles that may conceal preceding negative affect.

### Limitations and future directions

One of the limitations of the current study lies with the sampling. The sample was demographically limited, with mostly female and undergraduate students. Moreover, the sample size for this study might be underpowered for higher-order interactions, especially for the exploratory time-course analyses. Future studies should expand to a more culturally and demographically diverse sample, with increased sample size, to allow more complex analyses and more generalized conclusions to be made.

The second limitation concerns the experimental design. By using the between-participant design, although the perception of participants would not be affected by stimuli in another condition, we did not control for the individual differences between the participants when they made their own interpretations of fake smiles or genuine smiles. Future studies should also examine the within-participant differences in how attention is associated with the interpretation of smiles. Moreover, we did not directly examine participants’ rationale behind their ratings or their interpretations for the facial expression changes. Therefore, we lack the direct manipulation check for determining whether participants recognize the changes in facial expressions as a genuine reflection in the shift of emotions or as a masking of the preceding emotions. In addition, the current experimental design only explored the possible association but not any causal relationship between gaze behavior and subjective evaluations. Future studies could also explore in a clinical setting regarding the ability to read emotions from the changes in facial expressions.

The third limitation is regarding the stimuli design. First, the presentation of the 3-second still image of the final smile might strengthen the recency effects, biasing the subjective ratings to a more positive direction and potentially resulting in the absence of differences across conditions. Second, we only manipulated the temporal context preceding the smiles, which might not be sufficient for the participants to determine smiles following angry expressions as potentially masking smiles, as we did not detect reliable differences between conditions across all three ratings. The floor effect of anger also implies the possibility that the participants did not consider the posers with smiles to have an underlying anger. Future study should also increase the number of stimuli, provide the participants with more information on the social contexts and situations and less intense smiles to reduce the recency effect and increase ecological validity.

## Conclusions

The present study examined how a preceding temporal context (anger vs. neutral) shapes Japanese observers’ visual attention and subjective evaluations of dynamic smiles. The results suggested that ratings of sincerity, happiness and anger did not differ reliably between the two preceding contexts. However, there were significant attentional biases when viewing smiles changing from angry or neutral faces, with greater attention to the eye region in the anger-to-smile condition than in the neutral-to-smile condition. The results regarding the relationship between fixation duration to different facial parts and the ratings of the final smiles indicated that greater attention to the mouth was associated with increase in the perceived sincerity and happiness as well as decrease in the perceived anger. The time-course of changes in the gaze distribution emphasized important associations of subjective ratings and looking time to the mouth in the later phases of facial expression changes, however, these associations did not differ reliably by condition for all three ratings. Together, these results suggest that a potentially masking temporal context may elicit increased monitoring of the eyes, while final judgments may still be strongly associated with salient mouth cues and recent effects of the smiles—potentially reducing the perceived negativity from the preceding negative context. Future work should test these mechanisms using more naturalistic social contexts and stimuli, and by incorporating explicit authenticity-related judgments and broader contextual manipulations to investigate when and how masking interpretations of smiles emerge.

## Supporting information

S1 File Summary of the Pilot Study. (DOCX)

S1 Checklist Inclusivity in Global Research. (DOCX)

S1 Fig Mean Evaluations of the Final Smiles in Neutral-to-Smile Condition and Anger-to-Smile Condition. Red represents anger-to-smile condition and blue represents neutral-to-smile condition. Dots represent data points of each trial’s evaluation scores.
(TIF)

S1 Table Means, Minimums, Maximums and Ranges for Average Calibration and Validation Accuracy, Precision Standard Deviation (*SD*) and precision Root Mean Square (RMS) in Degree. (DOCX)

S2 Table LMM Results of the Effects of Condition, Stimulus’ Culture, Participant’s Gender and Stimulus’ Gender on Evaluations of the Final Smiles. The dependent variables are evaluations of sincerity, happiness and anger. The fixed effects included the main effects of condition (neutral-to-smile condition and anger-to-smile condition) and stimulus’ culture (Japanese and Chinese posers), and the main effects and interaction of participant’s gender and stimulus’ gender (Female and Male). Random effects included intercepts of the participant ID and stimulus media. Con in condition represents neutral-to-smile condition. Exp in condition represents anger-to-smile condition. CN in stimulus’ culture represents Chinese posers and JP represents Japanese posers. † *p* < .100.
(DOCX)

S3 Table Results of Ordinal Mixed Models of the Effects of Condition and Stimulus’ Culture on the Evaluations of Sincerity, Happiness and Anger. The ordinal mixed models for the evaluations of the final smiles were conducted as a supplementary robustness check for the LMM results. The dependent variables included evaluations of sincerity, happiness and anger, respectively. The predictors for each of the evaluation included condition (neutral-to-smile condition and anger-to-smile condition) and stimulus’ culture (Japanese and Chinese). OR stands for odds ratio. CI stands for confidence interval.
(DOCX)

S4 Table LMM Results of the Effects of AOI and Condition on Total Fixation Duration. The dependent variable was total fixation duration. The fixed effects included the main effects and interaction of AOI (eyes, nose, mouth) and condition (neutral-to-smile condition and anger-to-smile condition), and the main effect of stimulus’ culture (Japanese and Chinese posers). Random effects included intercepts of the participant ID and stimulus media. Con in condition represents neutral-to-smile condition. Exp in condition represents anger-to-smile condition. AOI terms reflect sum-to-zero coding and represent deviations from the grand mean; the mouth deviation is implied as −[Eyes + Nose]. * *p* < .050, *** *p* < .001.
(DOCX)

S5 Table LMM Results of the Effects of Condition, Time Bin, AOI, and Stimulus’ Culture on Fixation Duration Over Time. The dependent variable was time-binned fixation duration. Fixed effects included the main effects and interactions of condition (neutral-to-smile condition and anger-to-smile condition), time bin [1–9], AOI (eyes, nose, mouth), and the main effect of stimulus’ culture (Japanese and Chinese posers). Random effects included a participant ID random slope for time bin and a random intercept for stimulus media. Con in condition represents neutral-to-smile condition. Exp in condition represents anger-to-smile condition. CN in stimulus’ culture represents Chinese posers and JP represents Japanese posers. AOI terms reflect sum-to-zero coding and represent deviations from the grand mean; the mouth deviation is implied as −[Eyes + Nose]. ** *p* < .010, *** *p* < .001.
(DOCX)

S6 Table LMM Results of the Effects of Total Fixation Duration in Each AOI, Condition, Stimulus’ Culture on the Evaluations of the Final Smiles. The dependent variables were evaluations of sincerity, happiness and anger. The fixed effects included the main effects and interaction of AOI (eyes, nose, mouth) and condition (neutral-to-smile condition and anger-to-smile condition), and the main effects of stimulus’ culture (Japanese and Chinese posers) and total fixation duration to the face. Random effects included intercepts of the participant ID and stimulus media. The random intercept of stimulus media was dropped for evaluations of anger because stimulus media had extremely small variance. Con in condition represents neutral-to-smile condition. Exp in condition represents anger-to-smile condition. CN in stimulus’ culture represents Chinese posers and JP represents Japanese posers. † *p* < .100, * *p* < .050, ** *p* < .010.
(DOCX)

S7 Table LMM Results of the Effects of Binned Fixation Duration to the Eyes, Condition, Stimulus’ Culture and Binned Fixation Duration to the Face on the Evaluations of the Final Smiles. The dependent variable was evaluations of sincerity, happiness and anger. Fixed effects included the main effects and interactions of time-binned fixation duration to the eyes during each time bin (bin 1: early phase of morphing changes, bin 2: late phase of morphing changes, bin 3: static frames of final smiles) and condition (neutral-to-smile condition and anger-to-smile condition), and the main effect of stimulus’ culture (Japanese and Chinese posers) and time-binned fixation duration to the face during each time bin. Random effects included random intercepts for participant ID and stimulus media. Con in condition represents neutral-to-smile condition. Exp in condition represents anger-to-smile condition. CN in stimulus’ culture represents Chinese posers and JP represents Japanese posers. † *p* < .100.
(DOCX)

S8 Table LMM Results of the Effects of Binned Fixation Duration to the Mouth, Condition, Stimulus’ Culture and Binned Fixation Duration to the Face on the Evaluations of the Final Smiles. The dependent variable was evaluations of sincerity, happiness and anger. Fixed effects included the main effects and interactions of time-binned fixation duration to the mouth during each time bin (bin 1: early phase of morphing changes, bin 2: late phase of morphing changes, bin 3: static frames of final smiles) and condition (neutral-to-smile condition and anger-to-smile condition), and the main effect of stimulus’ culture (Japanese and Chinese posers) and time-binned fixation duration to the face during each time bin. Random effects included random intercepts for participant ID and stimulus media. Con in condition represents neutral-to-smile condition. Exp in condition represents anger-to-smile condition. CN in stimulus’ culture represents Chinese posers and JP represents Japanese posers. **†** *p* < .100, * *p* < .050, ** *p* < .010.
(DOCX)
