# Supplementary material for: Gaze allocation and evaluations of dynamic smiles following angry versus neutral expressions
Source: PLoS One. 2026 Aug 20;21(8):e0356283. doi: 10.1371/journal.pone.0356283 (PMC13492797; doi:10.1371/journal.pone.0356283)
Supplement: Supplementary file 1 [file pone.0356283.s012.pdf]

This study examined how visual attention and subjective judgments of dynamic simulations of smiling faces change when they transition from angry or neutral expressions. Additionally, it explored how attention allocation to different facial features affects subjective judgments of the final smiles. Finally, the study explored how the relationship between attention allocation and subjective ratings changes over time.

This is a very interesting and methodologically well-conducted study. I have only a few minor requests.

1) Could the authors indicate whether they assessed any gender differences and if there were differences when women evaluated female or male faces, and when men evaluated male or female faces?

2) It is not clear to me what the authors mean by "dynamic transition." The authors used a gradual-change paradigm?This aspect should be explained more clearly.

3) What are the implications of this study for facial emotion recognition? It might have been useful to assess the participants' ability in this area, for example by evaluating alexithymia. This aspect could be addressed within the limitations of the study.
