# Supplementary material for: Gaze allocation and evaluations of dynamic smiles following angry versus neutral expressions
Source: PLoS One. 2026 Aug 20;21(8):e0356283. doi: 10.1371/journal.pone.0356283 (PMC13492797; doi:10.1371/journal.pone.0356283)
Supplement: Supplementary file 2 [file pone.0356283.s001.docx]

**S1 File. Summary of the Pilot Study.**

We conducted an online pilot survey for the still images used to create morph movies in the experiment. For the pilot study, we used still images of six posers (three Japanese females and three Japanese males) from ATR Facial Expression Image Database (ATR-Promotions) and five posers (three Chinese males and two Chinese females) from intensity-classified Chinese facial emotion image database (Liu et al., 2019). The images included neutral, happy, angry and sad expressions. Each still image from the databases has already been validated by the database developers/providers for the intensities of the perceived emotions, such that the intended emotion received the highest intensity rating for each expression. However, to further validate the images by Chinese posers in the Japanese population, we asked 46 undergraduate students to evaluate the perceived emotions for a total of 44 images. For each image, participants were asked to rate the intensities of happiness, anger and sadness in a 10-point Likert-type scale (1: the least intensity – 10: the most intensity). We were able to confirm that, for all images of happy, angry and sad expressions, the participants rated the corresponding emotion as the most intense emotion (p<.001).

For the main experiment, we excluded one Chinese male poser, one Japanese male poser and one female Japanese poser either because they either had lowest mean ratings of sincerity or lowest mean ratings of happiness for their happy expressions. After selection, we had a total of eight posers, with two posers for each culture and each gender. All stimuli of angry expressions had mean ratings of anger above 5.13 and all stimuli of happy expressions had mean ratings of sincerity above 5.93 and mean ratings of happiness above 6.96.
