# Supplementary material for: Gaze allocation and evaluations of dynamic smiles following angry versus neutral expressions
Source: PLoS One. 2026 Aug 20;21(8):e0356283. doi: 10.1371/journal.pone.0356283 (PMC13492797; doi:10.1371/journal.pone.0356283)
Supplement: Supplementary file 4 [file pone.0356283.s004.docx]

**S1 Table. Means, Minimums, Maximums and Ranges for Average Calibration and Validation Accuracy, Precision Standard Deviation (*SD*) and precision Root Mean Square (RMS) in Degree.**

| **Metric** | **Mean** | **Min** | **Max** | **Range** |
| --- | --- | --- | --- | --- |
| Average calibration accuracy (degrees) | 1.10 | 0.25 | 1.95 | 1.7 |
| Average calibration precision SD (degrees) | 0.71 | 0.33 | 1.41 | 1.08 |
| Average calibration precision RMS (degrees) | 1.19 | 0.55 | 2.39 | 1.84 |
| Average validation accuracy (degrees) | 0.99 | 0.27 | 2.98 | 2.71 |
| Average validation precision SD (degrees) | 0.60 | 0.25 | 1.69 | 1.44 |
| Average validation precision RMS (degrees) | 0.57 | 0.2 | 1.22 | 1.02 |
