# Supplementary material for: Gaze allocation and evaluations of dynamic smiles following angry versus neutral expressions
Source: PLoS One. 2026 Aug 20;21(8):e0356283. doi: 10.1371/journal.pone.0356283 (PMC13492797; doi:10.1371/journal.pone.0356283)
Supplement: Supplementary file 5 [file pone.0356283.s005.docx]

**S2 Table. LMM Results of the Effects of Condition, Stimulus’ Culture, Participant’s Gender and Stimulus’ Gender on Evaluations of the Final Smiles.** The dependent variables are evaluations of sincerity, happiness and anger. The fixed effects included the main effects of condition (neutral-to-smile condition and anger-to-smile condition) and stimulus’ culture (Japanese and Chinese posers), and the main effects and interaction of participant’s gender and stimulus’ gender (Female and Male). Random effects included intercepts of the participant ID and stimulus media. Con in condition represents neutral-to-smile condition. Exp in condition represents anger-to-smile condition. CN in stimulus’ culture represents Chinese posers and JP represents Japanese posers. **†** *p* < .100.

| Fixed Effect | *b* | SE | 95% CI Lower | 95% CI Upper | *t* | df | *p* |  |
| --- | --- | --- | --- | --- | --- | --- | --- | --- |
| **Evaluation of Sincerity** |  |  |  |  |  |  |  |  |
| (Intercept) | 0.06 | 0.26 | -0.46 | 0.58 | 0.24 | 34 | 0.811 |  |
| Condition(Con-Exp) | -0.12 | 0.24 | -0.61 | 0.36 | -0.52 | 28 | 0.607 |  |
| Stimulus_culture(CN-JP) | 0.04 | 0.18 | -0.35 | 0.44 | 0.24 | 12 | 0.815 |  |
| Participant_gender(Female-Male) | -0.08 | 0.20 | -0.48 | 0.33 | -0.38 | 50 | 0.708 |  |
| Stimulus_gender(Female-Male) | -0.04 | 0.19 | -0.45 | 0.36 | -0.23 | 14 | 0.820 |  |
| Participant_gender(Female-Male):Stimulus_gender(Female-Male) | -0.03 | 0.11 | -0.23 | 0.18 | -0.25 | 317 | 0.803 |  |
| **Evaluation of Happiness** |  |  |  |  |  |  |  |  |
| (Intercept) | 0.18 | 0.28 | -0.40 | 0.76 | 0.64 | 35 | 0.529 |  |
| Condition(Con-Exp) | 0.16 | 0.27 | -0.38 | 0.70 | 0.60 | 29 | 0.553 |  |
| Stimulus_culture(CN-JP) | 0.08 | 0.20 | -0.37 | 0.52 | 0.37 | 12 | 0.718 |  |
| Participant_gender(Female-Male) | -0.26 | 0.21 | -0.69 | 0.17 | -1.22 | 50 | 0.229 |  |
| Stimulus_gender(Female-Male) | -0.03 | 0.21 | -0.47 | 0.42 | -0.12 | 13 | 0.906 |  |
| Participant_gender(Female-Male):Stimulus_gender(Female-Male) | -0.16 | 0.10 | -0.35 | 0.02 | -1.71 | 316 | 0.088 | † |
| **Evaluation of Anger** |  |  |  |  |  |  |  |  |
| (Intercept) | -0.04 | 0.13 | -0.30 | 0.22 | -0.33 | 46 | 0.745 |  |
| Condition(Con-Exp) | -0.12 | 0.11 | -0.35 | 0.11 | -1.07 | 42 | 0.292 |  |
| Stimulus_culture(CN-JP) | -0.11 | 0.06 | -0.23 | 0.02 | -1.90 | 12 | 0.082 | † |
| Participant_gender(Female-Male) | 0.06 | 0.13 | -0.20 | 0.31 | 0.45 | 51 | 0.656 |  |
| Stimulus_gender(Female-Male) | -0.02 | 0.06 | -0.16 | 0.11 | -0.35 | 19 | 0.727 |  |
| Participant_gender(Female-Male):Stimulus_gender(Female-Male) | 0.03 | 0.06 | -0.09 | 0.15 | 0.45 | 319 | 0.651 |  |
