# Supplementary material for: Gaze allocation and evaluations of dynamic smiles following angry versus neutral expressions
Source: PLoS One. 2026 Aug 20;21(8):e0356283. doi: 10.1371/journal.pone.0356283 (PMC13492797; doi:10.1371/journal.pone.0356283)
Supplement: Supplementary file 6 [file pone.0356283.s006.docx]

**S3 Table. Results of Ordinal Mixed Models of the Effects of Condition and Stimulus’ Culture on the Evaluations of Sincerity, Happiness and Anger.** The ordinal mixed models for the evaluations of the final smiles were conducted as a supplementary robustness check for the LMM results. The dependent variables are evaluations of sincerity, happiness and anger, respectively. The predictors for each of the evaluation included condition (neutral-to-smile condition and anger-to-smile condition) and stimulus’ culture (Japanese and Chinese). OR stands for odds ratio. CI stands for confidence interval.

| Dependent Variable | Predictor | OR | 95% CI | *z* | *p* |
| --- | --- | --- | --- | --- | --- |
| Sincerity | Condition | 0.93 | [0.58, 1.49] | -0.29 | 0.770 |
|  | Stimulus Culture | 1.04 | [0.73, 1.49] | 0.24 | 0.812 |
| Happiness | Condition | 1.22 | [0.66, 2.25] | 0.65 | 0.516 |
|  | Stimulus Culture | 1.06 | [0.67, 1.66] | 0.25 | 0.806 |
| Anger | Condition | 0.90 | [0.50, 1.60] | -0.37 | 0.713 |
|  | Stimulus Culture | 0.82 | [0.62, 1.09] | -1.35 | 0.176 |
|  | | | | | |
