# Supplementary material for: Gaze allocation and evaluations of dynamic smiles following angry versus neutral expressions
Source: PLoS One. 2026 Aug 20;21(8):e0356283. doi: 10.1371/journal.pone.0356283 (PMC13492797; doi:10.1371/journal.pone.0356283)
Supplement: Supplementary file 7 [file pone.0356283.s007.docx]

**S4 Table. LMM Results of the Effects of AOI and Condition on Total Fixation Duration.** The dependent variable was total fixation duration. The fixed effects included the main effects and interaction of AOI (eyes, nose, mouth) and condition (neutral-to-smile condition and anger-to-smile condition), and the main effect of stimulus’ culture (Japanese and Chinese posers). Random effects included intercepts of the participant ID and stimulus media. Con in condition represents neutral-to-smile condition. Exp in condition represents anger-to-smile condition. AOI terms reflect sum-to-zero coding and represent deviations from the grand mean; the mouth deviation is implied as −[Eyes + Nose]. * *p* < .050, *** *p* < .001.

| Fixed Effect | b | SE | 95% CI Lower | 95% CI Upper | *t* | df | *p* |  |
| --- | --- | --- | --- | --- | --- | --- | --- | --- |
| (Intercept) | -.01 | .06 | -.13 | .10 | -.23 | 42 | .819 |  |
| AOI (Eyes) | .48 | .04 | .41 | .55 | 13.30 | 1064 | <.001 | *** |
| AOI (Nose) | -.01 | .04 | -.08 | .06 | -.24 | 1064 | .812 |  |
| Condition (Con-Exp) | .05 | .06 | -.07 | .16 | .81 | 42 | .423 |  |
| Stimulus’ Culture (CN-JP) | .01 | .03 | -.06 | .08 | .40 | 13 | .699 |  |
| AOI (Eyes): Condition (Con-Exp) | -.09 | .04 | -.16 | -.02 | -2.51 | 1064 | .012 | * |
| AOI (Nose): Condition (Con-Exp) | .03 | .04 | -.04 | .10 | .91 | 1064 | .364 |  |
