# Supplementary material for: Gaze allocation and evaluations of dynamic smiles following angry versus neutral expressions
Source: PLoS One. 2026 Aug 20;21(8):e0356283. doi: 10.1371/journal.pone.0356283 (PMC13492797; doi:10.1371/journal.pone.0356283)
Supplement: Supplementary file 8 [file pone.0356283.s008.docx]

**S5 Table. LMM Results of the Effects of Condition, Time Bin, AOI, and Stimulus’ Culture on Fixation Duration Over Time.** The dependent variable was time-binned fixation duration. Fixed effects included the main effects and interactions of condition (neutral-to-smile condition and anger-to-smile condition), time bin (1-9), AOI (eyes, nose, mouth), and the main effect of stimulus’ culture (Japanese and Chinese posers). Random effects included a participant ID random slope for time bin and a random intercept for stimulus media. Con in condition represents neutral-to-smile condition. Exp in condition represents anger-to-smile condition. CN in stimulus’ culture represents Chinese posers and JP represents Japanese posers. AOI terms reflect sum-to-zero coding and represent deviations from the grand mean; the mouth deviation is implied as −[Eyes + Nose]. ** *p* < .010, *** *p* < .001.

| Fixed Effect | b | SE | 95% CI Lower | 95% CI Upper | *t* | df | *p* |  |
| --- | --- | --- | --- | --- | --- | --- | --- | --- |
| (Intercept) | 2.95 | .12 | 2.72 | 3.18 | 25.50 | 53 | < .001 | *** |
| Condition (Con-Exp) | .02 | .12 | -.21 | .26 | .21 | 53 | .838 |  |
| Time bin_c | .02 | .01 | -.01 | .04 | 1.26 | 50 | .215 |  |
| AOI (Eyes) | 1.37 | .03 | 1.30 | 1.43 | 40.40 | 11106 | < .001 | *** |
| AOI (Nose) | -.35 | .03 | -.42 | -.28 | -10.30 | 11106 | < .001 | *** |
| Stimulus_culture (CN-JP) | .05 | .03 | -.03 | .12 | 1.43 | 13 | .176 |  |
| Condition (Con-Exp):Time bin_c | .02 | .01 | -.01 | .04 | 1.56 | 50 | .126 |  |
| Condition (Con-Exp):AOI (Eyes) | -.20 | .03 | -.27 | -.14 | -6.00 | 11106 | < .001 | *** |
| Condition (Con-Exp):AOI (Nose) | .05 | .03 | -.02 | .11 | 1.40 | 11106 | .160 |  |
| Time bin_c:AOI (Eyes) | .04 | .01 | .01 | .06 | 2.93 | 11106 | .003 | ** |
| Time bin_c:AOI (Nose) | -.06 | .01 | -.09 | -.04 | -4.80 | 11106 | < .001 | *** |
| Condition (Con-Exp):Time bin_c:AOI (Eyes) | -.01 | .01 | -.04 | .01 | -1.00 | 11106 | .315 |  |
| Condition (Con-Exp):Time bin_c:AOI (Nose) | .00 | .01 | -.03 | .02 | -.07 | 11106 | .946 |  |
