# Supplementary material for: Gaze allocation and evaluations of dynamic smiles following angry versus neutral expressions
Source: PLoS One. 2026 Aug 20;21(8):e0356283. doi: 10.1371/journal.pone.0356283 (PMC13492797; doi:10.1371/journal.pone.0356283)
Supplement: Supplementary file 9 [file pone.0356283.s009.docx]

**S6 Table. LMM Results of the Effects of Total Fixation Duration in Each AOI, Condition, Stimulus’ Culture on the Evaluations of the Final Smiles.** The dependent variables were evaluations of sincerity, happiness and anger. The fixed effects included the main effects and interaction of AOI (eyes, nose, mouth) and condition (neutral-to-smile condition and anger-to-smile condition), and the main effects of stimulus’ culture (Japanese and Chinese posers) and total fixation duration to the face. Random effects included intercepts of the participant ID and stimulus media. The random intercept of stimulus media was dropped for evaluations of anger because stimulus media had extremely small variance. Con in condition represents neutral-to-smile condition. Exp in condition represents anger-to-smile condition. CN in stimulus’ culture represents Chinese posers and JP represents Japanese posers. **†** *p* < .100, * *p* < .050, ** *p* < .010.

| Fixed Effect | *b* | SE | 95% CI Lower | 95% CI Upper | *t* | df | *p* |  |
| --- | --- | --- | --- | --- | --- | --- | --- | --- |
| **Evaluation of Sincerity** |  |  |  |  |  |  |  |  |
| (Intercept) | 0.00 | 0.22 | -0.45 | 0.45 | 0.01 | 32 | 0.993 |  |
| logTFD_eyes_z | 0.00 | 0.15 | -0.29 | 0.29 | 0.01 | 351 | 0.989 |  |
| logTFD_nose_z | 0.17 | 0.15 | -0.12 | 0.46 | 1.17 | 364 | 0.241 |  |
| logTFD_mouth_z | 0.28 | 0.12 | 0.04 | 0.51 | 2.28 | 361 | 0.023 | * |
| Condition(Con-Exp) | -0.18 | 0.22 | -0.63 | 0.27 | -0.80 | 32 | 0.428 |  |
| Stimulus_culture(CN-JP) | 0.02 | 0.16 | -0.33 | 0.38 | 0.14 | 13 | 0.890 |  |
| logTFD_Face_z | -0.22 | 0.17 | -0.55 | 0.11 | -1.29 | 309 | 0.197 |  |
| logTFD_eyes_z:Condition(Con-Exp) | 0.07 | 0.12 | -0.17 | 0.31 | 0.59 | 302 | 0.555 |  |
| logTFD_nose_z:Condition(Con-Exp) | 0.17 | 0.14 | -0.09 | 0.44 | 1.26 | 362 | 0.207 |  |
| logTFD_mouth_z:Condition(Con-Exp) | -0.11 | 0.12 | -0.34 | 0.12 | -0.96 | 352 | 0.338 |  |
| **Evaluation of Happiness** |  |  |  |  |  |  |  |  |
| (Intercept) | 0.06 | 0.26 | -0.47 | 0.58 | 0.22 | 34 | 0.827 |  |
| logTFD_eyes_z | -0.10 | 0.14 | -0.37 | 0.17 | -0.73 | 357 | 0.465 |  |
| logTFD_nose_z | -0.01 | 0.14 | -0.28 | 0.26 | -0.06 | 356 | 0.955 |  |
| logTFD_mouth_z | 0.27 | 0.11 | 0.04 | 0.49 | 2.36 | 365 | 0.019 | * |
| Condition(Con-Exp) | 0.11 | 0.26 | -0.41 | 0.63 | 0.43 | 34 | 0.672 |  |
| Stimulus_culture(CN-JP) | 0.07 | 0.19 | -0.34 | 0.47 | 0.35 | 13 | 0.734 |  |
| logTFD_Face_z | -0.07 | 0.16 | -0.39 | 0.25 | -0.45 | 333 | 0.656 |  |
| logTFD_eyes_z:Condition(Con-Exp) | -0.04 | 0.12 | -0.27 | 0.18 | -0.36 | 327 | 0.720 |  |
| logTFD_nose_z:Condition(Con-Exp) | -0.19 | 0.13 | -0.44 | 0.06 | -1.49 | 361 | 0.136 |  |
| logTFD_mouth_z:Condition(Con-Exp) | 0.00 | 0.11 | -0.22 | 0.22 | -0.02 | 364 | 0.988 |  |
| **Evaluation of Anger** |  |  |  |  |  |  |  |  |
| (Intercept) | 0.00 | 0.11 | -0.21 | 0.22 | 0.05 | 49 | 0.964 |  |
| logTFD_eyes_z | -0.06 | 0.08 | -0.22 | 0.10 | -0.73 | 362 | 0.468 |  |
| logTFD_nose_z | -0.16 | 0.08 | -0.32 | 0.00 | -1.97 | 364 | 0.050 | * |
| logTFD_mouth_z | -0.22 | 0.07 | -0.35 | -0.09 | -3.33 | 366 | 0.001 | ** |
| Condition(Con-Exp) | -0.08 | 0.11 | -0.29 | 0.14 | -0.71 | 50 | 0.484 |  |
| Stimulus_culture(CN-JP) | -0.09 | 0.05 | -0.19 | 0.01 | -1.72 | 323 | 0.086 | † |
| logTFD_Face_z | 0.21 | 0.10 | 0.02 | 0.40 | 2.19 | 326 | 0.030 | * |
| logTFD_eyes_z:Condition(Con-Exp) | -0.03 | 0.07 | -0.17 | 0.10 | -0.48 | 319 | 0.633 |  |
| logTFD_nose_z:Condition(Con-Exp) | -0.15 | 0.07 | -0.30 | 0.00 | -2.02 | 367 | 0.044 | * |
| logTFD_mouth_z:Condition(Con-Exp) | 0.13 | 0.06 | 0.00 | 0.26 | 1.99 | 363 | 0.048 | * |
