# Supplementary material for: Gaze allocation and evaluations of dynamic smiles following angry versus neutral expressions
Source: PLoS One. 2026 Aug 20;21(8):e0356283. doi: 10.1371/journal.pone.0356283 (PMC13492797; doi:10.1371/journal.pone.0356283)
Supplement: Supplementary file 10 [file pone.0356283.s010.docx]

**S7 Table. LMM Results of the Effects of Binned Fixation Duration to the Eyes, Condition, Stimulus’ Culture and Binned Fixation Duration to the Face on the Evaluations of the Final Smiles.** The dependent variable was evaluations of sincerity, happiness and anger. Fixed effects included the main effects and interactions of time-binned fixation duration to the eyes during each time bin (bin 1: early phase of morphing changes, bin 2: late phase of morphing changes, bin 3: static frames of final smiles) and condition (neutral-to-smile condition and anger-to-smile condition), and the main effect of stimulus’ culture (Japanese and Chinese posers) and time-binned fixation duration to the face during each time bin. Random effects included random intercepts for participant ID and stimulus media. Con in condition represents neutral-to-smile condition. Exp in condition represents anger-to-smile condition. CN in stimulus’ culture represents Chinese posers and JP represents Japanese posers. **†** *p* < .100.

| Fixed Effect | *b* | SE | 95% CI Lower | 95% CI Upper | *t* | df | *p* |  |
| --- | --- | --- | --- | --- | --- | --- | --- | --- |
| **Evaluations of Sincerity** |  |  |  |  |  |  |  |  |
| (Intercept) | 0.02 | 0.23 | -0.45 | 0.49 | 0.08 | 31 | 0.940 |  |
| logTFD_Eyes_bin1_z | 0.21 | 0.14 | -0.05 | 0.48 | 1.56 | 363 | 0.119 |  |
| logTFD_Eyes_bin2_z | -0.28 | 0.15 | -0.58 | 0.03 | -1.80 | 352 | 0.073 | † |
| logTFD_Eyes_bin3_z | -0.15 | 0.15 | -0.44 | 0.14 | -1.02 | 362 | 0.309 |  |
| Condition(Con-Exp) | -0.14 | 0.23 | -0.61 | 0.33 | -0.60 | 31 | 0.551 |  |
| Stimulus_culture(CN-JP) | 0.06 | 0.17 | -0.31 | 0.44 | 0.36 | 13 | 0.723 |  |
| logTFD_Face_bin1_z | -0.07 | 0.15 | -0.36 | 0.23 | -0.44 | 354 | 0.662 |  |
| logTFD_Face_bin2_z | 0.07 | 0.14 | -0.21 | 0.34 | 0.48 | 341 | 0.634 |  |
| logTFD_Face_bin3_z | 0.02 | 0.16 | -0.30 | 0.34 | 0.14 | 360 | 0.886 |  |
| logTFD_Eyes_bin1_z:Condition(Con-Exp) | 0.03 | 0.13 | -0.23 | 0.29 | 0.23 | 363 | 0.821 |  |
| logTFD_Eyes_bin2_z:Condition(Con-Exp) | -0.09 | 0.14 | -0.36 | 0.18 | -0.64 | 352 | 0.521 |  |
| logTFD_Eyes_bin3_z:Condition(Con-Exp) | 0.19 | 0.13 | -0.07 | 0.46 | 1.46 | 358 | 0.144 |  |
| **Evaluations of Happiness** |  |  |  |  |  |  |  |  |
| (Intercept) | 0.06 | 0.26 | -0.48 | 0.59 | 0.21 | 32 | 0.834 |  |
| logTFD_Eyes_bin1_z | 0.16 | 0.13 | -0.09 | 0.41 | 1.24 | 360 | 0.214 |  |
| logTFD_Eyes_bin2_z | -0.28 | 0.14 | -0.56 | 0.00 | -1.95 | 347 | 0.052 | † |
| logTFD_Eyes_bin3_z | 0.01 | 0.14 | -0.26 | 0.29 | 0.11 | 359 | 0.916 |  |
| Condition(Con-Exp) | 0.14 | 0.26 | -0.39 | 0.67 | 0.53 | 32 | 0.600 |  |
| Stimulus_culture(CN-JP) | 0.10 | 0.20 | -0.33 | 0.52 | 0.48 | 13 | 0.638 |  |
| logTFD_Face_bin1_z | -0.10 | 0.14 | -0.38 | 0.18 | -0.71 | 359 | 0.481 |  |
| logTFD_Face_bin2_z | 0.12 | 0.13 | -0.13 | 0.37 | 0.95 | 334 | 0.341 |  |
| logTFD_Face_bin3_z | -0.02 | 0.15 | -0.32 | 0.28 | -0.14 | 359 | 0.885 |  |
| logTFD_Eyes_bin1_z:Condition(Con-Exp) | 0.06 | 0.12 | -0.18 | 0.30 | 0.49 | 361 | 0.622 |  |
| logTFD_Eyes_bin2_z:Condition(Con-Exp) | -0.07 | 0.13 | -0.32 | 0.19 | -0.53 | 348 | 0.599 |  |
| logTFD_Eyes_bin3_z:Condition(Con-Exp) | 0.17 | 0.12 | -0.08 | 0.41 | 1.36 | 354 | 0.175 |  |
| **Evaluations of Anger** |  |  |  |  |  |  |  |  |
| (Intercept) | -0.01 | 0.11 | -0.24 | 0.22 | -0.11 | 40 | 0.915 |  |
| logTFD_Eyes_bin1_z | -0.04 | 0.08 | -0.19 | 0.12 | -0.46 | 357 | 0.647 |  |
| logTFD_Eyes_bin2_z | 0.08 | 0.09 | -0.09 | 0.25 | 0.93 | 355 | 0.356 |  |
| logTFD_Eyes_bin3_z | 0.07 | 0.08 | -0.10 | 0.23 | 0.80 | 363 | 0.424 |  |
| Condition(Con-Exp) | -0.12 | 0.11 | -0.35 | 0.11 | -1.06 | 40 | 0.294 |  |
| Stimulus_culture(CN-JP) | -0.11 | 0.06 | -0.23 | 0.01 | -1.98 | 13 | 0.069 | † |
| logTFD_Face_bin1_z | -0.03 | 0.09 | -0.20 | 0.13 | -0.39 | 361 | 0.697 |  |
| logTFD_Face_bin2_z | -0.03 | 0.08 | -0.19 | 0.12 | -0.42 | 343 | 0.677 |  |
| logTFD_Face_bin3_z | 0.10 | 0.09 | -0.08 | 0.28 | 1.08 | 363 | 0.282 |  |
| logTFD_Eyes_bin1_z:Condition(Con-Exp) | -0.08 | 0.07 | -0.23 | 0.06 | -1.09 | 358 | 0.274 |  |
| logTFD_Eyes_bin2_z:Condition(Con-Exp) | 0.00 | 0.08 | -0.16 | 0.15 | -0.03 | 355 | 0.976 |  |
| logTFD_Eyes_bin3_z:Condition(Con-Exp) | -0.01 | 0.08 | -0.16 | 0.14 | -0.16 | 363 | 0.874 |  |
