# Supplementary material for: Gaze allocation and evaluations of dynamic smiles following angry versus neutral expressions
Source: PLoS One. 2026 Aug 20;21(8):e0356283. doi: 10.1371/journal.pone.0356283 (PMC13492797; doi:10.1371/journal.pone.0356283)
Supplement: Supplementary file 11 [file pone.0356283.s011.docx]

**S8 Table. LMM Results of the Effects of Binned Fixation Duration to the Mouth, Condition, Stimulus’ Culture and Binned Fixation Duration to the Face on the Evaluations of the Final Smiles.** The dependent variable was evaluations of sincerity, happiness and anger. Fixed effects included the main effects and interactions of time-binned fixation duration to the mouth during each time bin (bin 1: early phase of morphing changes, bin 2: late phase of morphing changes, bin 3: static frames of final smiles) and condition (neutral-to-smile condition and anger-to-smile condition), and the main effect of stimulus’ culture (Japanese and Chinese posers) and time-binned fixation duration to the face during each time bin. Random effects included random intercepts for participant ID and stimulus media. Con in condition represents neutral-to-smile condition. Exp in condition represents anger-to-smile condition. CN in stimulus’ culture represents Chinese posers and JP represents Japanese posers. **†** *p* < .100, * *p* < .050, ** *p* < .010.

| Fixed Effect | *b* | SE | 95% CI Lower | 95% CI Upper | *t* | df | *p* |  |
| --- | --- | --- | --- | --- | --- | --- | --- | --- |
| **Evaluations of Sincerity** |  |  |  |  |  |  |  |  |
| (Intercept) | 0.00 | 0.22 | -0.44 | 0.44 | 0.01 | 31 | 0.990 |  |
| logTFD_Mouth_bin1_z | -0.08 | 0.13 | -0.33 | 0.18 | -0.60 | 364 | 0.550 |  |
| logTFD_Mouth_bin2_z | 0.34 | 0.13 | 0.09 | 0.59 | 2.71 | 354 | 0.007 | ** |
| logTFD_Mouth_bin3_z | 0.23 | 0.12 | 0.00 | 0.45 | 1.96 | 364 | 0.050 | † |
| Condition(Con-Exp) | -0.19 | 0.22 | -0.63 | 0.25 | -0.88 | 31 | 0.386 |  |
| Stimulus_culture(CN-JP) | 0.00 | 0.16 | -0.34 | 0.34 | 0.01 | 13 | 0.993 |  |
| logTFD_Face_bin1_z | -0.04 | 0.15 | -0.32 | 0.25 | -0.27 | 355 | 0.786 |  |
| logTFD_Face_bin2_z | -0.10 | 0.12 | -0.34 | 0.15 | -0.78 | 342 | 0.434 |  |
| logTFD_Face_bin3_z | -0.09 | 0.14 | -0.36 | 0.19 | -0.60 | 356 | 0.549 |  |
| logTFD_Mouth_bin1_z:Condition(Con-Exp) | -0.34 | 0.13 | -0.59 | -0.09 | -2.66 | 366 | 0.008 | ** |
| logTFD_Mouth_bin2_z:Condition(Con-Exp) | 0.24 | 0.13 | -0.01 | 0.48 | 1.90 | 355 | 0.058 | † |
| logTFD_Mouth_bin3_z:Condition(Con-Exp) | -0.07 | 0.11 | -0.29 | 0.16 | -0.60 | 365 | 0.546 |  |
| **Evaluations of Happiness** |  |  |  |  |  |  |  |  |
| (Intercept) | 0.03 | 0.25 | -0.48 | 0.54 | 0.12 | 33 | 0.905 |  |
| logTFD_Mouth_bin1_z | -0.02 | 0.12 | -0.26 | 0.22 | -0.15 | 359 | 0.878 |  |
| logTFD_Mouth_bin2_z | 0.30 | 0.12 | 0.07 | 0.53 | 2.54 | 344 | 0.012 | * |
| logTFD_Mouth_bin3_z | 0.15 | 0.11 | -0.06 | 0.37 | 1.42 | 356 | 0.157 |  |
| Condition(Con-Exp) | 0.09 | 0.25 | -0.42 | 0.60 | 0.34 | 34 | 0.733 |  |
| Stimulus_culture(CN-JP) | 0.04 | 0.18 | -0.35 | 0.44 | 0.25 | 13 | 0.810 |  |
| logTFD_Face_bin1_z | -0.09 | 0.14 | -0.36 | 0.18 | -0.66 | 360 | 0.512 |  |
| logTFD_Face_bin2_z | -0.03 | 0.12 | -0.26 | 0.19 | -0.29 | 334 | 0.770 |  |
| logTFD_Face_bin3_z | -0.05 | 0.13 | -0.31 | 0.22 | -0.34 | 356 | 0.735 |  |
| logTFD_Mouth_bin1_z:Condition(Con-Exp) | -0.26 | 0.12 | -0.50 | -0.03 | -2.18 | 362 | 0.030 | * |
| logTFD_Mouth_bin2_z:Condition(Con-Exp) | 0.21 | 0.12 | -0.02 | 0.44 | 1.83 | 347 | 0.068 | † |
| logTFD_Mouth_bin3_z:Condition(Con-Exp) | 0.01 | 0.11 | -0.20 | 0.22 | 0.08 | 358 | 0.933 |  |
| **Evaluations of Anger** |  |  |  |  |  |  |  |  |
| (Intercept) | -0.01 | 0.10 | -0.22 | 0.20 | -0.10 | 48 | 0.919 |  |
| logTFD_Mouth_bin1_z | -0.04 | 0.07 | -0.18 | 0.10 | -0.57 | 365 | 0.569 |  |
| logTFD_Mouth_bin2_z | -0.04 | 0.07 | -0.17 | 0.10 | -0.54 | 347 | 0.589 |  |
| logTFD_Mouth_bin3_z | -0.21 | 0.06 | -0.33 | -0.08 | -3.21 | 363 | 0.001 | ** |
| Condition(Con-Exp) | -0.10 | 0.10 | -0.31 | 0.11 | -0.97 | 49 | 0.335 |  |
| Stimulus_culture(CN-JP) | -0.08 | 0.05 | -0.18 | 0.02 | -1.53 | 322 | 0.128 |  |
| logTFD_Face_bin1_z | -0.05 | 0.08 | -0.21 | 0.11 | -0.66 | 360 | 0.513 |  |
| logTFD_Face_bin2_z | 0.01 | 0.07 | -0.12 | 0.15 | 0.18 | 343 | 0.858 |  |
| logTFD_Face_bin3_z | 0.17 | 0.08 | 0.01 | 0.33 | 2.09 | 364 | 0.037 | * |
| logTFD_Mouth_bin1_z:Condition(Con-Exp) | 0.12 | 0.07 | -0.02 | 0.26 | 1.65 | 365 | 0.101 |  |
| logTFD_Mouth_bin2_z:Condition(Con-Exp) | 0.05 | 0.07 | -0.08 | 0.19 | 0.76 | 349 | 0.447 |  |
| logTFD_Mouth_bin3_z:Condition(Con-Exp) | -0.06 | 0.06 | -0.19 | 0.06 | -1.00 | 364 | 0.320 |  |
