# Supplementary material for: Gaze allocation and evaluations of dynamic smiles following angry versus neutral expressions
Source: PLoS One. 2026 Aug 20;21(8):e0356283. doi: 10.1371/journal.pone.0356283 (PMC13492797; doi:10.1371/journal.pone.0356283)
Supplement: Supplementary file 12 [file pone.0356283.s014.docx]

**Response to Reviewers**

**Title of the Paper:** Gaze allocation and evaluations of dynamic smiles following angry versus neutral expressions

**Paper Number:** PONE-D-26-07636

We thank the editor and reviewers for providing critical and constructive comments that have helped us enormously to improve the quality of our manuscript. Based on the comments, we have made significant modifications to the statistical analyses, especially for the time-course analyses. We have also modified the statements regarding time-varying gaze-evaluation associations throughout the main text so that the revised statements better and more clearly convey the research goal and conclusions of the current study. We have re-written the limitations section and expanded on the theoretical and practical implications of the current study in introduction and discussion sections. Our responses to each comment are shown below, together with how we revised the manuscript accordingly. We hope that our responses have appropriately addressed all concerns raised.

**Response to Journal Requirement #2:**

*Please include a complete copy of PLOS’ questionnaire on inclusivity in global research in your revised manuscript. Our policy for research in this area aims to improve transparency in the reporting of research performed outside of researchers’ own country or community. The policy applies to researchers who have travelled to a different country to conduct research, research with Indigenous populations or their lands, and research on cultural artefacts. The questionnaire can also be requested at the journal’s discretion for any other submissions, even if these conditions are not met. Please find more information on the policy and a link to download a blank copy of the questionnaire here: https://journals.plos.org/plosone/s/best-practices-in-research-reporting. Please upload a completed version of your questionnaire as Supporting Information when you resubmit your manuscript.*

**Response:**

Thank you for pointing out this issue. We have completed the questionnaire on inclusivity in global research and included the completed version as Supporting Information. In the manuscript, we have added a subsection of Inclusivity in global research under Materials and methods section as following.

(Page 17, line 359)

Additional information regarding the ethical, cultural, and scientific considerations specific to inclusivity in global research is included in the Supporting Information (S1 Checklist).

(Page 36, Line 785)

**S1 Checklist. Inclusivity in Global Research.**

**Response to Journal Requirement #3:**

*We note that the grant information you provided in the ‘Funding Information’ and ‘Financial Disclosure’ sections do not match.*

*When you resubmit, please ensure that you provide the correct grant numbers for the awards you received for your study in the ‘Funding Information’ section.*

**Response:**

Thank you for pointing out the discrepancy between the information in the Funding Information and Financial Disclosure sections. We have updated our statement in the Financial Disclosure section and updated the Cover Letter specifying this modification.

**Response to Journal Requirement #4:**

*We note that Figures 1 and 2 includes an image of a participant in the study.*

*As per the PLOS ONE policy (*[*http://journals.plos.org/plosone/s/submission-guidelines#loc-human-subjects-research*](http://journals.plos.org/plosone/s/submission-guidelines#loc-human-subjects-research)*) on papers that include identifying, or potentially identifying, information, the individual(s) or parent(s)/guardian(s) must be informed of the terms of the PLOS open-access (CC-BY) license and provide specific permission for publication of these details under the terms of this license. Please download the Consent Form for Publication in a PLOS Journal (*[*http://journals.plos.org/plosone/s/file?id=8ce6/plos-consent-form-english.pdf*](http://journals.plos.org/plosone/s/file?id=8ce6/plos-consent-form-english.pdf)*). The signed consent form should not be submitted with the manuscript, but should be securely filed in the individual's case notes. Please amend the methods section and ethics statement of the manuscript to explicitly state that the patient/participant has provided consent for publication: “The individual in this manuscript has given written informed consent (as outlined in PLOS consent form) to publish these case details”.*

*If you are unable to obtain consent from the subject of the photograph, you will need to remove the figure and any other textual identifying information or case descriptions for this individual.*

**Response:**

Thank you for pointing out the important issue regarding the figures. The individuals shown in Figures 1 and 2 were not participants in the current study but were posers of ATR Facial Expression Image Database and intensity-classified Chinese facial emotion image database. We have obtained permission from ATR-Promotions Inc. that granted us consent to use the stimuli (M02-f00-e00-SO-1.bmp and M02-f00-e00-AC-5.bmp) in the submitted manuscript under CC-BY license. We, therefore, amended Figure 2 to use only the stimuli that were granted with permission for publication. The main text and the captions of Figures 1 and 2 have been modified as following to explicitly state that we have obtained consent for publication under CC-BY license for the stimuli used in Figures 1 and 2.

(Page 10, Line 210)

Among the chosen facial expression images, two images from one Japanese poser were used in Figs 1 and 2 in the following sections. ATR Promotions, as the owner of the images, has given written permission to publish the two images under CC-BY license.

(Page 12, Line 256)

**Fig 1. Flow of procedures for one experimental trial.** For each one of the trials, the participants were first asked to fixate at a fixation cross, which was presented for 500 milliseconds, every time it appeared in the middle of the screen. Then, participants were asked to freely view the 9000-millisecond movie stimuli, which automatically started to play. Afterwards, participants were asked to press the number keys (0-9) to evaluate in a 10-point Likert-type scale about the ending smiles in terms of how sincere they think the final smiles were and the intensities of happiness, anger and sadness that they think the posers were feeling right now. The number key “0” means “10 (extremely)” in the evaluations, and the participants were briefed about it before the tasks started. Republished from ATR Facial Expression Image Database DB99 under a CC BY license, with permission from ATR-Promotions Inc., original copyright (2006).

(Page 13, Line 283)

**Fig 2. Examples of Areas of Interest (AOIs) for the Angry and Happy Expressions of a Japanese Male Poser.** AOI centers (left eye, right eye, nose and mouth) were first determined, and the average distance between each of the two adjacent AOI centers was computed as AOI span. The radii of the AOIs were determined to be 75% of the AOI span. AOIs were manually drawn using AOI tools in Tobii Pro Lab with the positions fixed based on corresponding AOI centers and the sizes determined by the radii of the AOIs. For adjacent AOIs that converged, the AOIs were divided along the perpendicular bisectors of the line segments connecting the two corresponding AOI centers. Dynamic AOIs were adjusted by defining two key frames, the starting and ending frames of the change, and creating the AOIs for each of the two key frames. The feature of dynamic AOI in Tobii Pro Lab automatically computed the intermediate changes between the two key frames. Republished from ATR Facial Expression Image Database DB99 under a CC BY license, with permission from ATR-Promotions Inc., original copyright (2006).

**Response to Editor’s Comments:**
**Overall Comment:**
*The general framing is promising with the strongest contribution of the paper in the combination of dynamic facial transitions, eye tracking, and time-binned gaze analyses. However, several claims need to be softened, and the statistical modeling requires clarification or re-analysis before one is confident that the reported gaze-evaluation effects are robust. More specifically:*
**Response:**

We greatly appreciate the editor’s critical comments. We have re-considered and modified the manuscript based on the important issues that you have pointed out, including our statements, statistical analyses and limitations of the current study. We hope that our modifications have appropriately addressed your concerns.

**Comment #1:**
*In the paper you repeatedly interpret the anger-to-smile condition as a "masking" context. You do acknowledge alternative interpretations such as emotion regulation, recovery, appeasement, or changed social intent, but several later claims still sound as if masking was actually manipulated or detected. This matters because the key behavioral result is that sincerity, happiness, and anger ratings did not differ significantly between conditions. The conclusion that temporal context "may elicit increased monitoring of the eyes" is supported, but claims about a "masking effect" or "hindering detection of fake smiles" should be reframed as mouth-attention positivity bias or reduced weighting of prior negative context, unless you add direct authenticity judgments or social-context manipulations.*

**Response:**

We thank the editor for pointing out this important issue, as the design of the current study did not directly examine the discrimination of facial expression masking. We have made the following changes in the main text accordingly to avoid confusions.

(Page 25, Line 541) in Discussion

The attention bias to a smiling mouth may be robust as it disrupts the recognition of the changes in the eye region regardless of the emotional valence. In the present study, despite the preceding expressions being negative or neutral, the unified changes in the facial features might enhance positive attention bias toward the smiling mouth and reduce weighting of the preceding negative contexts on the judgement of the final smiles.

(Page 29, Line 616) in Discussion

This result is consistent with the positive attention bias of the smiling mouth, as suggested by previous studies, on smile perception, in terms of reducing the weighting of incongruent contexts (8, 13, 28).

**Comment #2:**

*The repeated-measures structure is complex. Each participant sees multiple stimuli, each trial has multiple AOIs, and the time-course models expand each trial into AOI × time-bin observations. The models include random intercepts for participant and stimulus media, and one time-course model includes a participant random slope for time bin. However, for time-binned models predicting a single rating from many binned fixation rows, the same participant-by-stimulus rating appears to be reused across multiple rows. A random intercept for trial e.g. participant × stimulus/media, might needed to avoid treating repeated rows from the same trial as more independent than they are. Without this, standard errors for time-bin/AOI effects may be too small, especially for the reported time-varying mouth–anger association.*

**Response:**

This response is related to our response for Editor’s Comment #8, as they both concerned about the time-course analyses for the evaluations and the interpretations of the results. We thank the editor for pointing out these valid and crucial concerns. We agree that the interpretations of these models using repeated measures require caution because a single participant-by-media rating was represented across multiple AOI × time-bin rows. The reason why we were unable to incorporate the random intercept of (1 | Participant:Media) in the models is because the models could not converge.

To avoid using the higher-order interactions without accounting for the repeated measures in the model, and to more directly examine the second part of the fourth hypothesis, we have revised the statistical analyses for the time-varying visual attention and subjective evaluation association. Specifically, we no longer used the long-format AOI × time-bin model to predict a single rating. Instead, we restructured the data at the trial level, such that each participant × stimulus/media trial contributed a single row to each rating model. For each trial, we divided the movie stimuli into early morphing phases (bin 1: 0-3 s), late morphing phases (bin 2: 3-6 s), and final phase of static smiles (6-9 s). We fitted the LMM models for each rating, with binned fixation duration to the eyes in each time bin and their interactions with condition as fixed effect, as well as stimulus’ culture and binned fixation duration to the face in each time bin as covariates. Considering that both previous studies and the results of the current study suggest that gaze allocation to the mouth is also important when judging smiles, we conducted additional exploratory analyses using binned fixation duration to the mouth in each time bin in the models. We found that, although the results had changed from the previous analyses, the associations between gaze allocation to the mouth region in the later phases of the facial expression changes and the subjective evaluations of the final smiles were still observed. Therefore, we argue that the key conclusions and implications of the current study that could be inferred were largely intact. We hope that the modifications could address the concern for the robustness of the analyses.

As stated above, the modifications were made throughout the main text. Below were some examples of the modifications that we made in the methods and results sections. The modifications regarding the hypothesis, discussion and conclusion sections were shown in our response to Editor’s Comment #8.

(Page 15, Line 330) in Methods

For the second half of the fourth hypothesis examining the relationship between ratings of the final smiles and the time course of fixation durations to the eyes, we first divided the movie stimuli into three consecutive time bins of 3000 milliseconds. The first time bin included frames of early morphing phase, the second time bin included frames of late morphing changes, and the third time bin included the static frames of the final expressions. We then fitted LMMs for each of the ratings, with the main effects and interactions of binned fixation duration to the eyes in each time bin and condition, the main effects of fixation duration to the face in each time bin as fixed effects, and with random intercepts for participant (1 | Participant) and stimulus media (1 | Media). In addition, as an exploratory analysis, to examine the relationship between subjective evaluations and time course of visual attention to the mouth, we fitted the LMMs for each rating using binned fixation duration to the mouth, condition, and their interactions as fixed effects.

(Page 21, line 441) in Results

To examine time-varying associations between gaze allocation and subjective ratings, we first performed separate LMM analyses for each of the evaluations (sincerity, happiness, and anger), with the main effects and interactions of condition and fixation duration to the eyes in early morphing phase (bin 1), late morphing phase (bin 2) and final static phase (bin 3), including stimulus’ culture and fixation duration to the face in each time bin as covariates

…(Please see the manuscript for the full details of the results)

The results indicated that during the presentation of static final smiles, participants who fixated longer to the mouth would rate the smiles as less angry. Across all models (S7-S8 Tables), condition did not reliably moderate the fixation–evaluation slopes or their change across time bins, suggesting that the time-varying fixation–evaluation associations were broadly similar between the anger-to-smile and neutral-to-smile conditions.

(Page 39, Line 833) in Supporting information

**S7 Table. LMM Results of the Effects of Binned Fixation Duration to the Eyes, Condition, Stimulus’ Culture and Binned Fixation Duration to the Face on the Evaluations of the Final Smiles.** The dependent variable was evaluations of sincerity, happiness and anger. Fixed effects included the main effects and interactions of time-binned fixation duration to the eyes during each time bin (bin 1: early phase of morphing changes, bin 2: late phase of morphing changes, bin 3: static frames of final smiles) and condition (neutral-to-smile condition and anger-to-smile condition), and the main effect of stimulus’ culture (Japanese and Chinese posers) and time-binned fixation duration to the face during each time bin. Random effects included random intercepts for participant ID and stimulus media. Con in condition represents neutral-to-smile condition. Exp in condition represents anger-to-smile condition. CN in stimulus’ culture represents Chinese posers and JP represents Japanese posers. † p < .100.

**S8 Table. LMM Results of the Effects of Binned Fixation Duration to the Mouth, Condition, Stimulus’ Culture and Binned Fixation Duration to the Face on the Evaluations of the Final Smiles.** The dependent variable was evaluations of sincerity, happiness and anger. Fixed effects included the main effects and interactions of time-binned fixation duration to the mouth during each time bin (bin 1: early phase of morphing changes, bin 2: late phase of morphing changes, bin 3: static frames of final smiles) and condition (neutral-to-smile condition and anger-to-smile condition), and the main effect of stimulus’ culture (Japanese and Chinese posers) and time-binned fixation duration to the face during each time bin. Random effects included random intercepts for participant ID and stimulus media. Con in condition represents neutral-to-smile condition. Exp in condition represents anger-to-smile condition. CN in stimulus’ culture represents Chinese posers and JP represents Japanese posers. † p < .100, * p < .050, ** p < .010.

**Comment #3:**

*You mention that condition and stimulus culture were not significant for sincerity, happiness, and anger. That supports the claim "we did not detect differences", not "the conditions do not differ". This is especially important because the power analysis was for medium between-group effects, while the paper's main theoretical claims involve interactions, gaze–rating associations, and time-binned effects. If you want to argue that dynamic context alone is insufficient to affect evaluations, you should report effect estimates and confidence intervals for the condition effects and ideally include equivalence tests or Bayesian evidence for the null. Otherwise, the conclusion should read more cautiously.*

**Response:**

We thank the editor for pointing out the issue with the conclusion and the lack of reporting for the statistical values in the results. We have modified the conclusion as follows and reported the effect estimates and confidence intervals for the condition effects of all three ratings (sincerity, happiness and anger) in the main text as following. We have also included the report of confidence intervals throughout the results section.

(Page 30, Line 653) in Discussion

Second, we only manipulated the temporal context preceding the smiles, which might not be sufficient for the participants to determine smiles following angry expressions as potentially masking smiles, as we did not detect differences between conditions across all three ratings.

(Page 18, Line 380) in Results

Condition (sincerity: *b* = -.12, SE = .24, 95% CI [-.61, .36], *p* = .607; happiness: *b* = .14, SE = .26, 95% CI [-.39, .68], *p* = .586; anger: *b* = -.12, SE = .11, 95% CI [-.34, .11], *p* = .296), stimulus’ culture, participant’s and stimulus’ gender were not significant for all models.

**Comment #4:**

*The descriptive means for anger are very low, e.g. 1.53 in the neutral-to-smile condition and 1.81 in the anger-to-smile condition on a 10-point scale. This raises concern about skew and floor effects. A standard Gaussian LMM may be acceptable as a sensitivity analysis, but you should show residual diagnostics or consider ordinal/robust mixed models. This is particularly important because several key findings involve anger ratings.*

**Response:**

We thank the editor for pointing out the important issue. We have run the residual diagnostics and subsequently the ordinal mixed models for ratings of sincerity, happiness and anger. We have reported the results of the residual diagnostics in the main text as follows. We have also added S3 Table to include the results for the ordinal mixed models. The discussion regarding the floor effect observed for the ratings of anger was included in the limitation section.

(Page 14, Line 302) in Methods

According to the results of the residual diagnostics for all three ratings, we observed ceiling effects for the ratings of sincerity (*p* = .003) and happiness (*p* = .017) as well as a floor effect for the rating of anger (*p* < .001). To account for the skewness of the data, we also fitted the ordinal mixed models for each of the ratings, with condition and stimulus’ culture as predictors and with the same random effects as in LMMs.

(Page 18, Line 384) in Results

The results of ordinal mixed models (see S3 Table) for all three ratings were also consistent with those from LMMs.

(Page 30, Line 656) in Discussion

The floor effect of anger also implies the possibility that the participants did not consider the posers with smiles to have an underlying anger.

(Page 37, Line 798) in Supporting information

**S3 Table. Results of Ordinal Mixed Models of the Effects of Condition and Stimulus’ Culture on the Evaluations of Sincerity, Happiness and Anger.** The ordinal mixed models for the evaluations of the final smiles were conducted as a supplementary robustness check for the LMM results. The dependent variables included evaluations of sincerity, happiness and anger, respectively. The predictors for each of the evaluation included condition (neutral-to-smile condition and anger-to-smile condition) and stimulus’ culture (Japanese and Chinese). OR stands for odds ratio. CI stands for confidence interval.

**Comment #5:**

*More time looking at the mouth generally means less time available for eyes/nose, and total looking time differs across participants and trials. You log-transform and z-score fixation durations, but the manuscript does not explain how zero fixations were handled, which is crucial for log transformation. You should clarify whether you used log(x), log(x+1), or another transformation. You should also consider using fixation proportions, total face-looking time as a covariate, or compositional approaches, because interpreting mouth fixation as an independent predictor can otherwise be misleading.*

**Response:**

Thank you for pointing out the lack of explanation on the details regarding log transformation. We have used log(x+1) transformation for fixation duration and modified the main text in the following way specifying this detail.

We have also conducted additional analyses by adding total fixation duration to the face as covariate. There were significant main effects of fixation duration to the mouth for all three ratings as well as significant interactions with condition for the fixation duration to nose and mouth for the ratings of anger. The detailed results are reported in S6 Table.

(Page 16, Line 345) in Methods

Also, the total fixation duration and binned fixation duration were normalized via log-transformation using log(x+1) function and standardized into z-scores.

(Page 15, Line 314) in Methods

…we fitted LMMs for each of the ratings with total fixation duration to each AOI, condition and their interactions as well as stimulus’ culture and total fixation duration to the face as fixed effects…

(Page 20, Line 423) in Results

There was a significant main effect of fixation duration to the mouth for the evaluations of sincerity (*b* = .28, SE = .12, 95% CI [.04, .51], *p* = .023), happiness (*b* = .27, SE = .11, 95% CI [.04, .49], *p* = .019) and anger (*b* = -.22, SE= .07, 95% CI [-.35, -.09], *p* = .001), revealing that participants who fixated longer to the mouth rated the final smiles as more sincere, happier and less angry. Also, for the evaluations of anger, there was a significant main effect of fixation duration to the nose (*b* = -.16, SE= .08, 95% CI [-.32, .00], *p* = .050), indicating that participants who fixated longer to the nose also rated the smiles as less angry. The total fixation to the face was significant as well (*b* = .21, SE= .10, 95% CI [.02, .40], *p* = .030), indicating that participants who overall fixated longer during the stimulus presentation rated the smiles as angrier. There were also significant interactions of fixation duration to the nose and Condition (b = -.15, SE= .07, 95% CI [-.30, .00], *p* = .044), and of fixation duration to the mouth and Condition (*b* = .13, SE= .06, 95% CI [.00, .26], *p* = .048). Simple slopes revealed that participants in the anger-to-smile condition who fixated longer to the mouth rated the final smiles as less angry (estimate = -.35, SE = .08, 95% CI [-.58, -.19], *p* < .001), whereas fixation duration to the nose in the neutral-to-smile condition was associated with less perceived anger (estimate = -.31, SE = .14, 95% CI [-.58, -.04], *p* = .048).

(Page 25, Line 526) in Discussion

We also found a negative association between fixation duration to the nose and perceived anger in the neutral-to-smile condition. The nose region affords a holistic overview of the face, and in the absence of a preceding angry context, greater holistic sampling may have reinforced perception of the final expression as a coherent genuine smile, thereby lowering attributions of anger.

(Page 38, Line 823) in Supporting information

**S6 Table. LMM Results of the Effects of Total Fixation Duration in Each AOI, Condition, Stimulus’ Culture on the Evaluations of the Final Smiles.**…and the main effects of stimulus’ culture (Japanese and Chinese posers) and total fixation duration to the face.

**Comment #6:**

*The paper sometimes says attention "affected" perceived anger or that mouth attention had an "impact". But the design is observational with respect to gaze as participants were not experimentally assigned to look at the mouth or eyes. The reported effects show associations, not causal effects. For example, longer mouth fixation was associated with higher sincerity/happiness and lower anger ratings, and in the anger-to-smile condition longer mouth fixation predicted lower anger. I would recommended using alterative wording such as "was associated with, was linked to, or may index greater reliance on mouth cues" instead.*

**Response:**

Thank you for pointing out the confusing phrasings used in the main text. We have made corresponding changes throughout the main text. Below is an example for the changes that we have made.

(Page 29, Line 613) in Discussion

Based on the results of the exploratory analyses, increased attention to the smiling mouth, when it became salient during the later phase of changing facial expressions, was linked strongly to higher perceived sincerity and happiness as well as lower perceived anger.

**Comment #7:**

*The sample is adequate for a basic between-condition comparison but limited for the more complex mixed models. You should acknowledge that the sample is a culturally and demographically narrow sample, mostly female and undergraduate, and that the study may be underpowered for higher-order interactions. Additionally, the stimulus design should be described more explicitly. It is important to state clearly whether the final happy/smile image was identical across the anger-to-smile and neutral-to-smile versions for each poser. If not, condition effects may be confounded with smile intensity or stimulus identity. The manuscript says there were 8 anger-to-smile movies, 8 neutral-to-smile movies, and 8 fillers, each with a 6-second morph and 3-second final still image. That is useful, but pairing/counterbalancing needs to be unmistakable. Moreover, the 3-second static final expression likely strengthens recency effects and may explain the absence of rating differences. You discuss this possibility, noting that participants may have focused on the high-intensity final smiles rather than the initial angry expressions. This should be elevated from a discussion point to a central design limitation. Finally, eye-tracking quality metrics should be added including calibration accuracy, track loss, trial exclusions, off-AOI looking, and whether AOI fixation durations sum to total face or screen fixation time.*

**Response:**

We thank the editor for pointing out the limitations and/or the lack of descriptions regarding the sampling, stimulus design, pairing and counterbalancing, and the eye-tracking quality metrics. These comments helped us improve our manuscript and more clearly specify the limitations of the current study. We respond to each of the issues raised in the comment as following.

1) We agree with editor’s comments regarding the sampling of the present study being demographically narrow, the sample size being underpowered for complex interactions, and the 3-second still image of the final smiles strengthening the recency effects. We have included these limitations in the main text.

(Page 29, Line 631) in Discussion

The sample was demographically limited, with mostly female and undergraduate students. Moreover, the sample size for this study might be underpowered for higher-order interactions, especially for the exploratory time-course analyses.

(Page 30, Line 650) in Discussion

First, the presentation of the 3-second still image of the final smile might strengthen the recency effects, biasing the subjective ratings to a more positive direction and potentially resulting in the absence of differences across conditions.

2) We thank the editor for pointing out the lack of description regarding the stimulus design. The final smile images were identical across the two conditions for each poser. We have modified the main text including the description as follows.

(Page 10, Line 217) in Methods

The final smile was identical across different conditions for each poser.

3) We agree with the editor that the pairing and counterbalancing of the stimuli need more detailed description. We have modified the main text as follows.

(Page 10, Line 214) in Methods

…we created 24 morph movie stimuli (9-second-long with 135 frames), with 8 (2 posers × 2 stimulus’ cultures × 2 stimulus’ genders) for the anger-to-smile condition, 8 (same as above) for the neutral-to-smile condition, and 8 (same as above) for the smile-to-sadness filler tasks.

(Page 11, Line 225) in Methods

The participants were randomly assigned to either the anger-to-smile condition or the neutral-to-smile condition. They were presented with the stimuli of each corresponding condition as well as the stimuli of filler tasks, in a randomized order.

4) We thank the editor for pointing out the lack of reports of eye-tracking quality measures. We have added the reports in the methods section as follows.

(Page 9, Line 188) in Methods

In addition, we excluded 9.13% (38 out of 416) of the trials due to insufficient looking time.

(Page 13, Line 278) in Methods

The sum of total fixation duration to the AOIs (eyes, nose and mouth) is on average 17 milliseconds less than the total fixation duration to the screen. The eye-tracking metrics of calibration and validation accuracy and precision are reported in S1 Table.

(Page 36, Line 782) in Supporting information

**S1 Table. Means, Minimums, Maximums and Ranges for Average Calibration and Validation Accuracy, Precision Standard Deviation (*SD*) and precision Root Mean Square (RMS) in Degree.**

**Comment #8:**
*Time-course analyses showed AOI and AOI × condition effects, plus time-bin × AOI effects, but not the predicted condition × time-bin × AOI interaction. Therefore, the manuscript should avoid saying that the anger context changed the trajectory of gaze over time. It changed the distribution of gaze across AOIs, while the time trajectory did not differ reliably by condition. Also, the mouth-fixation association with ratings is plausible but should be treated cautiously. The reported mouth effects for sincerity, happiness, and anger are statistically significant, but some p-values are modest, and the time-varying anger interaction is p = .047. Given multiple models and multiple outcomes, you should either correct/qualify these analyses or explicitly label them exploratory.*
**Response:**

We again thank the editor for providing important suggestions on improving the manuscript regarding the time-course analyses and statements regarding the results of those analyses. For the first part of the comments, we have carefully revised the manuscript so that our statements describe the results more appropriately. For the second part of the comments regarding the time-varying gaze-evaluation associations, as stated in our response to Editor’s Comment #2, we have modified the analyses so that they examine the hypothesis more directly and do not require higher order interactions that the current study design might not be sufficient for. The second part of the hypothesis mainly concerns the time-varying eye-evaluation association. However, given the results from both previous studies and the current studies, we considered that examining the time-varying mouth-evaluation association could expand the findings of the current study and decided to conduct additional exploratory analyses for it. Overall, the associations with time-varying gaze allocation to the mouth were observed for all three ratings, however, we still did not observe reliable interactions with condition across all three ratings. Therefore, the overall conclusions and implications did not change greatly.

We have updated the manuscript accounting for the changes in the results. The modifications regarding the methods and results were shown in our response for Editor’s Comment #2. Below are the modifications made in abstract, introduction, discussion and conclusion sections.

(Page 2, Line 34) in Abstract

Time-course analyses for gaze distribution over time indicated that gaze shifted from the nose toward the eyes and mouth over time and exploratory analyses also showed that the temporal changes in the gaze distribution in later phases of morphing changes were associated with subjective ratings. However, these associations did not reliably differ by preceding emotional contexts.

(Page 8, Line 168) in Introduction

As previous studies also emphasize the importance of attention to the mouth region when evaluating genuine smiles, we would also conduct exploratory analyses on the relationship between subjective evaluations and time course of visual attention to the mouth.

(Page 26, Line 560) in Discussion

The second half of the fourth hypothesis regarding the association between the ratings of the final smiles and the attention to the eyes in the early phase was not supported, as we only observed marginally significant main effects for the binned fixation duration to the eyes and did not observe any interactions with condition. In the exploratory LMM models for the binned fixation duration to the mouth, we instead observed that positive associations for the perceived sincerity and happiness, especially in the neutral-to-smile condition and an overall negative association for the perceived anger. These associations were observed in later morphing phases when the smiles were more prominent, or the presentation of static final smiles.

(Page 31, Line 672) in Conclusions

The time-course of changes in the gaze distribution emphasized important associations of subjective ratings and looking time to the mouth in the later phases of facial expression changes, however, these associations did not differ reliably by condition for all three ratings.

**Comment #9:**
*The limitations section is useful but incomplete. You acknowledge the between-participant design and the lack of explicit social context, but you should also add the limited sample diversity, small number of posers/stimuli, possible floor effects for anger, possible recency effects due to the 3-second final still image, absence of direct authenticity/masking manipulation checks, non-causal gaze–rating associations, and uncertainty about the robustness of time-binned models. Please discuss.*

**Response:**

We thank the editor for providing constructive advice for the limitations section to help improve the manuscript. We acknowledge all the limitations pointed out and have rewritten the limitation sections as follows. Part of the modified limitation was also based on Comment #3 from Reviewer #2.

We thank the Editor for this important clarification. We revised the manuscript to distinguish between the overall distribution of gaze across AOIs and changes in the temporal trajectory of gaze. The retained time-course analysis showed AOI × Condition and Time bin × AOI interactions, whereas the predicted Condition × Time bin × AOI interaction was not significant. We therefore no longer state that the preceding emotional context changed the trajectory of gaze over time. Instead, we describe the result as a condition-related difference in the overall distribution of gaze across facial regions.

In addition, following the concern raised in Comment #2 and Comment #8, we have revised the analyses examining time-varying gaze-evaluation associations, the updated results did not show consistent and reliable differences between conditions for all three ratings. We have revised the manuscript accordingly, removing the models involving the repeated-measure structure and subsequent post-hoc analyses.

Below are the modifications we made to the abstract and limitation sections reflecting the changes.

(Page 2, Line 41) in Abstract

In the present paradigm, the preceding facial expression did not produce detectable differences in explicit evaluations; whether such temporal information contributes to authenticity inferences remains to be tested directly. Future research should examine this question using explicit authenticity judgments and richer social-context cues that motivate masking.

(Page 29, Line 631) in Discussion

One of the limitations of the current study lies with the sampling. The sample was demographically limited, with mostly female and undergraduate students. Moreover, the sample size for this study might be underpowered for higher-order interactions, especially for the exploratory time-course analyses. Future studies should expand to a more culturally and demographically diverse sample, with increased sample size, to allow more complex analyses and more generalized conclusions to be made.

The second limitation concerns the experimental design. By using the between-participant design, although the perception of participants would not be affected by stimuli in another condition, we did not control for the individual differences between the participants when they made their own interpretations of fake smiles or genuine smiles. Future studies should also examine the within-participant differences in how attention is associated with the interpretation of smiles. Moreover, we did not directly examine participants’ rationale behind their ratings or their interpretations for the facial expression changes. Therefore, we lack the direct manipulation check for determining whether participants recognize the changes in facial expressions as a genuine reflection in the shift of emotions or as a masking of the preceding emotions. In addition, the current experimental design only explored the possible association but not any causal relationship between gaze behavior and subjective evaluations. Future studies could also explore in a clinical setting regarding the ability to read emotions from the changes in facial expressions.

The third limitation is regarding the stimuli design. First, the presentation of the 3-second still image of the final smile might strengthen the recency effects, biasing the subjective ratings to a more positive direction and potentially resulting in the absence of differences across conditions. Second, we only manipulated the temporal context preceding the smiles, which might not be sufficient for the participants to determine smiles following angry expressions as potentially masking smiles, as we did not detect reliable differences between conditions across all three ratings. The floor effect of anger also implies the possibility that the participants did not consider the posers with smiles to have an underlying anger. It is important to provide the participants with more information on the social contexts and situations and less intense smiles to reduce the recency effect and increase ecological validity.

**Response to Reviewer #1’s Comments:
General Comment:**

*The study is very well written and makes a clear and interesting contribution. The introduction sets up the aims well, the literature review is strong, the hypotheses are clear, the methods and analyses are sound, and the figures are easy to interpret. Overall, this is a well executed and clearly presented study. I would support publication after minor revisions.*

*My suggestions are listed below:*
**Response:**

We thank Reviewer #1 for providing us with valuable suggestions in highlighting the importance and contribution of this study. Our responses are as follows.

**Comment #1:**
*The Introduction could end with a brief statement on the theoretical and/or practical importance of the study. This would help emphasize why the work matters beyond the immediate findings.*

**Response:**

We thank Reviewer #1 for suggesting that we add a statement on the importance of the study. We have modified the introduction section as follows.

(Page 8, Line 176) in Introduction

In doing so, this study highlights that the integration and processing of contextual information in emotional perception might differ in terms of gaze behavior and evaluations.

**Comment #2:**
*The Discussion could more clearly highlight the broader importance of the study for social cognition. Because faces and facial expressions are central to everyday social interaction, you could say more about how the findings help us understand the interpretation of changing facial expressions in real-life interactions.*
**Comment #3:**

*The Discussion could also more clearly explain the study’s theoretical contribution. In particular, the findings seem to distinguish between gaze behaviour and explicit evaluations. This is interesting because it suggests that attention to facial information and later social-affective judgments may not reflect the same process.*
**Comment #4:**

*Relatedly, you may consider whether the difference between gaze behaviour and explicit ratings is not only a difference between perceptual and evaluative processing, but also a difference in the nature of the measures themselves. Eye movements are relatively spontaneous and less consciously controlled, whereas ratings of sincerity, happiness, and anger are explicit judgments and may be shaped by reflection or social norms. This may help explain why gaze allocation differed between conditions whereas the final ratings did not.*

**Response:**

This response is for Comment #2-4 of Reviewer #1 and Comment #3 for Reviewer #2. We thank both reviewers for suggesting that we should include further implications and contributions as it amplifies the impact of the present study. We have modified the discussion section of the main text corresponding to the comments as following.

(Page 27, Line 579) in Discussion

Moreover, the discrepancies between in how gaze allocation and evaluations differ by preceding emotional expressions suggest that the spontaneous eye movements and subjective social-emotional evaluations reflect different processes. As in real-life, how we perceive dynamically conflicting and consistent facial expressions might require us to pay more attention to the most salient part or the more rapidly changing facial features. Therefore, the visual attention may be shaped more in a bottom-up and less consciously controlled way. On the other hand, making social-affective inferences about smile sincerity or the current emotional state might require one to integrate surrounding information in a more top-down way, influencing by personal beliefs, cultural norms or social-emotional situations. We are also interested in examining how people perceive mixed emotions from the changes in facial expressions, as cultural experiences and norms regarding the socially appropriate emotional expressions are likely to shape how people perceive and recognize emotions.

**Response to Reviewer #2’s Comments:**

**General Comment:**

*I believe the article is well-written and employs an appropriate methodological approach and sound statistical analysis. Since the topic is important to general psychology, it would be helpful to provide additional details about the methodology and results, as well as discuss the broader implications of the findings.*

*This study examined how visual attention and subjective judgments of dynamic simulations of smiling faces change when they transition from angry or neutral expressions. Additionally, it explored how attention allocation to different facial features affects subjective judgments of the final smiles. Finally, the study explored how the relationship between attention allocation and subjective ratings changes over time.*

*This is a very interesting and methodologically well-conducted study. I have only a few minor requests.*

**Response:**

We thank Reviewer #2 for providing valuable comments to help us further improve our manuscript. Our responses to the comments are as follows.

**Comment #1:**

*Could the authors indicate whether they assessed any gender differences and if there were differences when women evaluated female or male faces, and when men evaluated male or female faces?*

**Response:**

We thank Reviewer #2 for bringing up this interesting topic. We have conducted additional analyses for each of the evaluations, by adding the interaction term of participant’s gender and stimulus’ gender in the model. We did not detect gender differences in the subjective evaluations. However, the results should be interpreted with caution, considering the sampling in the current study is biased with mostly female participants. We modified the main text as following and updated S2 Table for this revision.

(Page 14, Line 298) in Methods

For the first hypothesis regarding the evaluation of final smiles, we fitted linear mixed-effect models (LMMs) for each of the evaluations (sincerity, happiness and anger) with the main effects of condition and stimulus’ culture and with the main effects and interaction of participant’s gender and stimulus’ gender as fixed effects and random intercepts of participant ID (1 | Participant) and stimulus media (1 | Media).

(Page 18, Line 382) in Results

…stimulus’ culture, participant’s and stimulus’ gender were not significant for all models. The detailed results are reported in S2 Table…

(Page 37, Line 789) in Supporting information

**S2 Table. LMM Results of the Effects of Condition, Stimulus’ Culture, Participant’s Gender and Stimulus’ Gender on Evaluations of the Final Smiles.** The dependent variables are evaluations of sincerity, happiness and anger. The fixed effects included the main effects of condition (neutral-to-smile condition and anger-to-smile condition) and stimulus’ culture (Japanese and Chinese posers), and the main effects and interaction of participant’s gender and stimulus’ gender (Female and Male). Random effects included intercepts of the participant ID and stimulus media. Con in condition represents neutral-to-smile condition. Exp in condition represents anger-to-smile condition. CN in stimulus’ culture represents Chinese posers and JP represents Japanese posers. **†** *p* < .100.

**Comment #2:**

*It is not clear to me what the authors mean by "dynamic transition." The authors used a gradual change paradigm? This aspect should be explained more clearly.*

**Response:**

Thank you for pointing out the lack of explanation in the dynamic aspect of the stimuli. Using morphing software, we first determined the localizers for the key features of the face for both the starting frame (neutral or angry expression) and ending frame (smiles). Then, the software automatically calculated and outputted continuous changes from the starting point to the ending point. We have modified the main text as following to avoid the confusion.

(Page 11, Line 223) in Methods

We determined the localizers for the key features of the faces, and the software computed the linear and continuous changes of the corresponding localizers from the starting to ending frames at the speed of 15 frames per second.

**Comment #3:**

*What are the implications of this study for facial emotion recognition? It might have been useful to assess the participants' ability in this area, for example by evaluating alexithymia. This aspect could be addressed within the limitations of the study.*

Response:

We thank Reviewer #2 for pointing out the lack of discussion regarding the practical implications of the current study. We have modified the discussion and limitation sections accordingly. The modifications are also shown in our responses to Editor’s Comment #9 and Reviewer #1’s Comment #2-4.

(Page 30, Line 647) in Discussion

Future studies could also explore in a clinical setting regarding the ability to read emotions from the changes in facial expressions.

We also modified the main text to fix a typo as following.

(Page 25, Line 537) in Discussion

Moreover, the result that ~~g~~ greater attention to the mouth was associated with lower perceived anger in the anger-to-smile condition is in line with the results from Iwasaki and Noguchi (13)…
