# Supplementary material for: Gaze allocation and evaluations of dynamic smiles following angry versus neutral expressions
Source: PLoS One. 2026 Aug 20;21(8):e0356283. doi: 10.1371/journal.pone.0356283 (PMC13492797; doi:10.1371/journal.pone.0356283)
Supplement: Peer review history

# Peer review: Decision Letter 0

**DOI:** 10.1371/journal.pone.0356283.r001

**Authors:** Domna Banakou

12 May 2026

Dear Dr. Wu,

Thank you for submitting your manuscript to PLOS ONE. After careful consideration, we feel that it has merit but does not fully meet PLOS ONE’s publication criteria as it currently stands. Therefore, we invite you to submit a revised version of the manuscript that addresses the points raised during the review process.

The work is not simply another genuine-vs-fake smile discrimination study and its value is in asking whether **temporal context**  and **time-resolved gaze**  **allocation**  shape evaluations of smiles. Overall, the manuscript is timely and well-written but it still needs some major revisions regarding the methodology and reporting of the findings before it can be considered further. Please review the detailed comments (Editor and Reviewers) below.

Please submit your revised manuscript by Jun 26 2026 11:59PM. If you will need more time than this to complete your revisions, please reply to this message or contact the journal office at plosone@plos.org. When you're ready to submit your revision, log on to https://www.editorialmanager.com/pone/ and select the 'Submissions Needing Revision' folder to locate your manuscript file.

- A letter that responds to each point raised by the academic editor and reviewer(s). You should upload this letter as a separate file labeled 'Response to Reviewers'.
- A marked-up copy of your manuscript that highlights changes made to the original version. You should upload this as a separate file labeled 'Revised Manuscript with Track Changes'.
- An unmarked version of your revised paper without tracked changes. You should upload this as a separate file labeled 'Manuscript'.

If you would like to make changes to your financial disclosure, please include your updated statement in your cover letter. Guidelines for resubmitting your figure files are available below the reviewer comments at the end of this letter.

If applicable, we recommend that you deposit your laboratory protocols in protocols.io to enhance the reproducibility of your results. Protocols.io assigns your protocol its own identifier (DOI) so that it can be cited independently in the future. For instructions see: https://journals.plos.org/plosone/s/submission-guidelines#loc-laboratory-protocols. Additionally, PLOS ONE offers an option for publishing peer-reviewed Lab Protocol articles, which describe protocols hosted on protocols.io. Read more information on sharing protocols at https://plos.org/protocols?utm_medium=editorial-email&utm_source=authorletters&utm_campaign=protocols.

As the corresponding author, your ORCID iD is verified in the submission system and will appear in the published article. PLOS supports the use of ORCID, and we encourage all coauthors to register for an ORCID iD and use it as well. Please encourage your coauthors to verify their ORCID iD within the submission system before final acceptance, as unverified ORCID iDs will not appear in the published article. *Only*  the individual author can complete the verification step; PLOS staff *cannot*  verify ORCID iDs on behalf of authors.

We look forward to receiving your revised manuscript.

Kind regards,

Domna Banakou

Academic Editor

PLOS One

Journal Requirements:

When submitting your revision, we need you to address these additional requirements.

1. Please ensure that your manuscript meets PLOS ONE's style requirements, including those for file naming. The PLOS ONE style templates can be found at

https://journals.plos.org/plosone/s/file?id=wjVg/PLOSOne_formatting_sample_main_body.pdf and

https://journals.plos.org/plosone/s/file?id=ba62/PLOSOne_formatting_sample_title_authors_affiliations.pdf

2. Please include a complete copy of PLOS’ questionnaire on inclusivity in global research in your revised manuscript. Our policy for research in this area aims to improve transparency in the reporting of research performed outside of researchers’ own country or community. The policy applies to researchers who have travelled to a different country to conduct research, research with Indigenous populations or their lands, and research on cultural artefacts. The questionnaire can also be requested at the journal’s discretion for any other submissions, even if these conditions are not met.  Please find more information on the policy and a link to download a blank copy of the questionnaire here: https://journals.plos.org/plosone/s/best-practices-in-research-reporting. Please upload a completed version of your questionnaire as Supporting Information when you resubmit your manuscript.

3. We note that the grant information you provided in the ‘Funding Information’ and ‘Financial Disclosure’ sections do not match.

When you resubmit, please ensure that you provide the correct grant numbers for the awards you received for your study in the ‘Funding Information’ section.

4. We note that Figures 1 and 2 includes an image of a participant in the study.

As per the PLOS ONE policy (http://journals.plos.org/plosone/s/submission-guidelines#loc-human-subjects-research) on papers that include identifying, or potentially identifying, information, the individual(s) or parent(s)/guardian(s) must be informed of the terms of the PLOS open-access (CC-BY) license and provide specific permission for publication of these details under the terms of this license. Please download the Consent Form for Publication in a PLOS Journal (http://journals.plos.org/plosone/s/file?id=8ce6/plos-consent-form-english.pdf). The signed consent form should not be submitted with the manuscript, but should be securely filed in the individual's case notes. Please amend the methods section and ethics statement of the manuscript to explicitly state that the patient/participant has provided consent for publication: “The individual in this manuscript has given written informed consent (as outlined in PLOS consent form) to publish these case details”.

If you are unable to obtain consent from the subject of the photograph, you will need to remove the figure and any other textual identifying information or case descriptions for this individual.

5. If the reviewer comments include a recommendation to cite specific previously published works, please review and evaluate these publications to determine whether they are relevant and should be cited. There is no requirement to cite these works unless the editor has indicated otherwise.

Additional Editor Comments:

The general framing is promising with the strongest contribution of the paper in the combination of dynamic facial transitions, eye tracking, and time-binned gaze analyses. However, several claims need to be softened, and the statistical modeling requires clarification or re-analysis before one is confident that the reported gaze-evaluation effects are robust. More specifically:

1. In the paper you repeatedly interpret the anger-to-smile condition as a "masking" context. You do acknowledge alternative interpretations such as emotion regulation, recovery, appeasement, or changed social intent, but several later claims still sound as if masking was actually manipulated or detected. This matters because the key behavioral result is that sincerity, happiness, and anger ratings did not differ significantly between conditions. The conclusion that temporal context "may elicit increased monitoring of the eyes" is supported, but claims about a "masking effect" or "hindering detection of fake smiles" should be reframed as mouth-attention positivity bias or reduced weighting of prior negative context, unless you add direct authenticity judgments or social-context manipulations.

2. The repeated-measures structure is complex. Each participant sees multiple stimuli, each trial has multiple AOIs, and the time-course models expand each trial into AOI × time-bin observations. The models include random intercepts for participant and stimulus media, and one time-course model includes a participant random slope for time bin. However, for time-binned models predicting a single rating from many binned fixation rows, the same participant-by-stimulus rating appears to be reused across multiple rows. A random intercept for trial e.g. participant × stimulus/media, might needed to avoid treating repeated rows from the same trial as more independent than they are. Without this, standard errors for time-bin/AOI effects may be too small, especially for the reported time-varying mouth–anger association.

3. You mention that condition and stimulus culture were not significant for sincerity, happiness, and anger. That supports the claim "we did not detect differences", not "the conditions do not differ". This is especially important because the power analysis was for medium between-group effects, while the paper's main theoretical claims involve interactions, gaze–rating associations, and time-binned effects. If you want to argue that dynamic context alone is insufficient to affect evaluations, you should report effect estimates and confidence intervals for the condition effects and ideally include equivalence tests or Bayesian evidence for the null. Otherwise, the conclusion should read more cautiously.

4. The descriptive means for anger are very low, e.g. 1.53 in the neutral-to-smile condition and 1.81 in the anger-to-smile condition on a 10-point scale. This raises concern about skew and floor effects. A standard Gaussian LMM may be acceptable as a sensitivity analysis, but you should show residual diagnostics or consider ordinal/robust mixed models. This is particularly important because several key findings involve anger ratings.

5. More time looking at the mouth generally means less time available for eyes/nose, and total looking time differs across participants and trials. You log-transform and z-score fixation durations, but the manuscript does not explain how zero fixations were handled, which is crucial for log transformation. You should clarify whether you used log(x), log(x+1), or another transformation. You should also consider using fixation proportions, total face-looking time as a covariate, or compositional approaches, because interpreting mouth fixation as an independent predictor can otherwise be misleading.

6. The paper sometimes says attention "affected" perceived anger or that mouth attention had an "impact". But the design is observational with respect to gaze as participants were not experimentally assigned to look at the mouth or eyes. The reported effects show associations, not causal effects. For example, longer mouth fixation was associated with higher sincerity/happiness and lower anger ratings, and in the anger-to-smile condition longer mouth fixation predicted lower anger. I would recommended using alterative wording such as "was associated with, was linked to, or may index greater reliance on mouth cues" instead.

7. The sample is adequate for a basic between-condition comparison but limited for the more complex mixed models. You should acknowledge that the sample is a culturally and demographically narrow sample, mostly female and undergraduate, and that the study may be underpowered for higher-order interactions. Additionally, the stimulus design should be described more explicitly. It is important to state clearly whether the final happy/smile image was identical across the anger-to-smile and neutral-to-smile versions for each poser. If not, condition effects may be confounded with smile intensity or stimulus identity. The manuscript says there were 8 anger-to-smile movies, 8 neutral-to-smile movies, and 8 fillers, each with a 6-second morph and 3-second final still image. That is useful, but pairing/counterbalancing needs to be unmistakable. Moreover, the 3-second static final expression likely strengthens recency effects and may explain the absence of rating differences. You discuss this possibility, noting that participants may have focused on the high-intensity final smiles rather than the initial angry expressions. This should be elevated from a discussion point to a central design limitation. Finally, eye-tracking quality metrics should be added including calibration accuracy, track loss, trial exclusions, off-AOI looking, and whether AOI fixation durations sum to total face or screen fixation time.

8. Time-course analyses showed AOI and AOI × condition effects, plus time-bin × AOI effects, but not the predicted condition × time-bin × AOI interaction. Therefore, the manuscript should avoid saying that the anger context changed the trajectory of gaze over time. It changed the distribution of gaze across AOIs, while the time trajectory did not differ reliably by condition. Also, the mouth-fixation association with ratings is plausible but should be treated cautiously. The reported mouth effects for sincerity, happiness, and anger are statistically significant, but some p-values are modest, and the time-varying anger interaction is p = .047. Given multiple models and multiple outcomes, you should either correct/qualify these analyses or explicitly label them exploratory.

9. The limitations section is useful but incomplete. You acknowledge the between-participant design and the lack of explicit social context, but you should also add the limited sample diversity, small number of posers/stimuli, possible floor effects for anger, possible recency effects due to the 3-second final still image, absence of direct authenticity/masking manipulation checks, non-causal gaze–rating associations, and uncertainty about the robustness of time-binned models. Please discuss.

[Note: HTML markup is below. Please do not edit.]

Reviewers' comments:

Reviewer's Responses to Questions

**Comments to the Author**

1. Is the manuscript technically sound, and do the data support the conclusions?

Reviewer #1: Yes

Reviewer #2: Yes

**********

2. Has the statistical analysis been performed appropriately and rigorously?

Reviewer #1: Yes

Reviewer #2: Yes

**********

3. Have the authors made all data underlying the findings in their manuscript fully available?

The PLOS Data policy

Reviewer #1: Yes

Reviewer #2: Yes

**********

4. Is the manuscript presented in an intelligible fashion and written in standard English?

Reviewer #1: Yes

Reviewer #2: Yes

**********

Reviewer #1: The study is very well written and makes a clear and interesting contribution. The introduction sets up the aims well, the literature review is strong, the hypotheses are clear, the methods and analyses are sound, and the figures are easy to interpret. Overall, this is a well executed and clearly presented study. I would support publication after minor revisions.

My suggestions are listed below:

• The Introduction could end with a brief statement on the theoretical and/or practical importance of the study. This would help emphasize why the work matters beyond the immediate findings.

• The Discussion could more clearly highlight the broader importance of the study for social cognition. Because faces and facial expressions are central to everyday social interaction, you could say more about how the findings help us understand the interpretation of changing facial expressions in real-life interactions.

• The Discussion could also more clearly explain the study’s theoretical contribution. In particular, the findings seem to distinguish between gaze behaviour and explicit evaluations. This is interesting because it suggests that attention to facial information and later social-affective judgments may not reflect the same process.

• Relatedly, you may consider whether the difference between gaze behaviour and explicit ratings is not only a difference between perceptual and evaluative processing, but also a difference in the nature of the measures themselves. Eye movements are relatively spontaneous and less consciously controlled, whereas ratings of sincerity, happiness, and anger are explicit judgments and may be shaped by reflection or social norms. This may help explain why gaze allocation differed between conditions whereas the final ratings did not.

Reviewer #2: I believe the article is well-written and employs an appropriate methodological approach and sound statistical analysis. Since the topic is important to general psychology, it would be helpful to provide additional details about the methodology and results, as well as discuss the broader implications of the findings.

**********

what does this mean?). If published, this will include your full peer review and any attached files.

If you choose “no”, your identity will remain anonymous but your review may still be made public.

**Do you want your identity to be public for this peer review?**  For information about this choice, including consent withdrawal, please see our Privacy Policy

Reviewer #1: **Yes:** Özge Karakale

Reviewer #2: **Yes:** Maria Casagrande

**********

[NOTE: If reviewer comments were submitted as an attachment file, they will be attached to this email and accessible via the submission site. Please log into your account, locate the manuscript record, and check for the action link "View Attachments". If this link does not appear, there are no attachment files.]

To ensure your figures meet our technical requirements, please review our figure guidelines: https://journals.plos.org/plosone/s/figures

You may also use PLOS’s free figure tool, NAAS, to help you prepare publication quality figures: https://journals.plos.org/plosone/s/figures#loc-tools-for-figure-preparation.

NAAS will assess whether your figures meet our technical requirements by comparing each figure against our figure specifications.

Attachment Submitted filename: Rev PlosOne 20.04.2026.pdf

# Author comment: Author response to Decision Letter 1

**DOI:** 10.1371/journal.pone.0356283.r002

10 Jul 2026

We thank the editor and reviewers for providing critical and constructive comments that have helped us enormously to improve the quality of our manuscript. Based on the comments, we have made significant modifications to the statistical analyses, especially for the time-course analyses. We have also modified the statements regarding time-varying gaze-evaluation associations throughout the main text so that the revised statements better and more clearly convey the research goal and conclusions of the current study. We have re-written the limitations section and expanded on the theoretical and practical implications of the current study in introduction and discussion sections. Our responses to each comment are shown below, together with how we revised the manuscript accordingly. We hope that our responses have appropriately addressed all concerns raised.

Response to Editor’s Comments:

Overall Comment:

The general framing is promising with the strongest contribution of the paper in the combination of dynamic facial transitions, eye tracking, and time-binned gaze analyses. However, several claims need to be softened, and the statistical modeling requires clarification or re-analysis before one is confident that the reported gaze-evaluation effects are robust. More specifically:

Response:

We greatly appreciate the editor’s critical comments. We have re-considered and modified the manuscript based on the important issues that you have pointed out, including our statements, statistical analyses and limitations of the current study. We hope that our modifications have appropriately addressed your concerns.

Comment #1:

In the paper you repeatedly interpret the anger-to-smile condition as a "masking" context. You do acknowledge alternative interpretations such as emotion regulation, recovery, appeasement, or changed social intent, but several later claims still sound as if masking was actually manipulated or detected. This matters because the key behavioral result is that sincerity, happiness, and anger ratings did not differ significantly between conditions. The conclusion that temporal context "may elicit increased monitoring of the eyes" is supported, but claims about a "masking effect" or "hindering detection of fake smiles" should be reframed as mouth-attention positivity bias or reduced weighting of prior negative context, unless you add direct authenticity judgments or social-context manipulations.

Response:

We thank the editor for pointing out this important issue, as the design of the current study did not directly examine the discrimination of facial expression masking. We have made the following changes in the main text accordingly to avoid confusions.

(Page 25, Line 541) in Discussion

The attention bias to a smiling mouth may be robust as it disrupts the recognition of the changes in the eye region regardless of the emotional valence. In the present study, despite the preceding expressions being negative or neutral, the unified changes in the facial features might enhance positive attention bias toward the smiling mouth and reduce weighting of the preceding negative contexts on the judgement of the final smiles.

(Page 29, Line 616) in Discussion

This result is consistent with the positive attention bias of the smiling mouth, as suggested by previous studies, on smile perception, in terms of reducing the weighting of incongruent contexts (8, 13, 28).

Comment #2:

The repeated-measures structure is complex. Each participant sees multiple stimuli, each trial has multiple AOIs, and the time-course models expand each trial into AOI × time-bin observations. The models include random intercepts for participant and stimulus media, and one time-course model includes a participant random slope for time bin. However, for time-binned models predicting a single rating from many binned fixation rows, the same participant-by-stimulus rating appears to be reused across multiple rows. A random intercept for trial e.g. participant × stimulus/media, might needed to avoid treating repeated rows from the same trial as more independent than they are. Without this, standard errors for time-bin/AOI effects may be too small, especially for the reported time-varying mouth–anger association.

Response:

This response is related to our response for Editor’s Comment #8, as they both concerned about the time-course analyses for the evaluations and the interpretations of the results. We thank the editor for pointing out these valid and crucial concerns. We agree that the interpretations of these models using repeated measures require caution because a single participant-by-media rating was represented across multiple AOI × time-bin rows. The reason why we were unable to incorporate the random intercept of (1 | Participant:Media) in the models is because the models could not converge.

To avoid using the higher-order interactions without accounting for the repeated measures in the model, and to more directly examine the second part of the fourth hypothesis, we have revised the statistical analyses for the time-varying visual attention and subjective evaluation association. Specifically, we no longer used the long-format AOI × time-bin model to predict a single rating. Instead, we restructured the data at the trial level, such that each participant × stimulus/media trial contributed a single row to each rating model. For each trial, we divided the movie stimuli into early morphing phases (bin 1: 0-3 s), late morphing phases (bin 2: 3-6 s), and final phase of static smiles (6-9 s). We fitted the LMM models for each rating, with binned fixation duration to the eyes in each time bin and their interactions with condition as fixed effect, as well as stimulus’ culture and binned fixation duration to the face in each time bin as covariates. Considering that both previous studies and the results of the current study suggest that gaze allocation to the mouth is also important when judging smiles, we conducted additional exploratory analyses using binned fixation duration to the mouth in each time bin in the models. We found that, although the results had changed from the previous analyses, the associations between gaze allocation to the mouth region in the later phases of the facial expression changes and the subjective evaluations of the final smiles were still observed. Therefore, we argue that the key conclusions and implications of the current study that could be inferred were largely intact. We hope that the modifications could address the concern for the robustness of the analyses.

As stated above, the modifications were made throughout the main text. Below were some examples of the modifications that we made in the methods and results sections. The modifications regarding the hypothesis, discussion and conclusion sections were shown in our response to Editor’s Comment #8.

(Page 15, Line 330) in Methods

For the second half of the fourth hypothesis examining the relationship between ratings of the final smiles and the time course of fixation durations to the eyes, we first divided the movie stimuli into three consecutive time bins of 3000 milliseconds. The first time bin included frames of early morphing phase, the second time bin included frames of late morphing changes, and the third time bin included the static frames of the final expressions. We then fitted LMMs for each of the ratings, with the main effects and interactions of binned fixation duration to the eyes in each time bin and condition, the main effects of fixation duration to the face in each time bin as fixed effects, and with random intercepts for participant (1 | Participant) and stimulus media (1 | Media). In addition, as an exploratory analysis, to examine the relationship between subjective evaluations and time course of visual attention to the mouth, we fitted the LMMs for each rating using binned fixation duration to the mouth, condition, and their interactions as fixed effects.

(Page 21, line 441) in Results

To examine time-varying associations between gaze allocation and subjective ratings, we first performed separate LMM analyses for each of the evaluations (sincerity, happiness, and anger), with the main effects and interactions of condition and fixation duration to the eyes in early morphing phase (bin 1), late morphing phase (bin 2) and final static phase (bin 3), including stimulus’ culture and fixation duration to the face in each time bin as covariates

…(Please see the manuscript for the full details of the results)

The results indicated that during the presentation of static final smiles, participants who fixated longer to the mouth would rate the smiles as less angry. Across all models (S7-S8 Tables), condition did not reliably moderate the fixation–evaluation slopes or their change across time bins, suggesting that the time-varying fixation–evaluation associations were broadly similar between the anger-to-smile and neutral-to-smile conditions.

(Page 39, Line 833) in Supporting information

S7 Table. LMM Results of the Effects of Binned Fixation Duration to the Eyes, Condition, Stimulus’ Culture and Binned Fixation Duration to the Face on the Evaluations of the Final Smiles. The dependent variable was evaluations of sincerity, happiness and anger. Fixed effects included the main effects and interactions of time-binned fixation duration to the eyes during each time bin (bin 1: early phase of morphing changes, bin 2: late phase of morphing changes, bin 3: static frames of final smiles) and condition (neutral-to-smile condition and anger-to-smile condition), and the main effect of stimulus’ culture (Japanese and Chinese posers) and time-binned fixation duration to the face during each time bin. Random effects included random intercepts for participant ID and stimulus media. Con in condition represents neutral-to-smile condition. Exp in condition represents anger-to-smile condition. CN in stimulus’ culture represents Chinese posers and JP represents Japanese posers. † p < .100.

S8 Table. LMM Results of the Effects of Binned Fixation Duration to the Mouth, Condition, Stimulus’ Culture and Binned Fixation Duration to the Face on the Evaluations of the Final Smiles. The dependent variable was evaluations of sincerity, happiness and anger. Fixed effects included the main effects and interactions of time-binned fixation duration to the mouth during each time bin (bin 1: early phase of morphing changes, bin 2: late phase of morphing changes, bin 3: static frames of final smiles) and condition (neutral-to-smile condition and anger-to-smile condition), and the main effect of stimulus’ culture (Japanese and Chinese posers) and time-binned fixation duration to the face during each time bin. Random effects included random intercepts for participant ID and stimulus media. Con in condition represents neutral-to-smile condition. Exp in condition represents anger-to-smile condition. CN in stimulus’ culture represents Chinese posers and JP represents Japanese posers. † p < .100, * p < .050, ** p < .010.

Comment #3:

You mention that condition and stimulus culture were not significant for sincerity, happiness, and anger. That supports the claim "we did not detect differences", not "the conditions do not differ". This is especially important because the power analysis was for medium between-group effects, while the paper's main theoretical claims involve interactions, gaze–rating associations, and time-binned effects. If you want to argue that dynamic context alone is insufficient to affect evaluations, you should report effect estimates and confidence intervals for the condition effects and ideally include equivalence tests or Bayesian evidence for the null. Otherwise, the conclusion should read more cautiously.

Response:

We thank the editor for pointing out the issue with the conclusion and the lack of reporting for the statistical values in the results. We have modified the conclusion as follows and reported the effect estimates and confidence intervals for the condition effects of all three ratings (sincerity, happiness and anger) in the main text as following. We have also included the report of confidence intervals throughout the results section.

(Page 30, Line 653) in Discussion

Second, we only manipulated the temporal context preceding the smiles, which might not be sufficient for the participants to determine smiles following angry expressions as potentially masking smiles, as we did not detect differences between conditions across all three ratings.

(Page 18, Line 380) in Results

Condition (sincerity: b = -.12, SE = .24, 95% CI [-.61, .36], p = .607; happiness: b = .14, SE = .26, 95% CI [-.39, .68], p = .586; anger: b = -.12, SE = .11, 95% CI [-.34, .11], p = .296), stimulus’ culture, participant’s and stimulus’ gender were not significant for all models.

Comment #4:

The descriptive means for anger are very low, e.g. 1.53 in the neutral-to-smile condition and 1.81 in the anger-to-smile condition on a 10-point scale. This raises concern about skew and floor effects. A standard Gaussian LMM may be acceptable as a sensitivity analysis, but you should show residual diagnostics or consider ordinal/robust mixed models. This is particularly important because several key findings involve anger ratings.

Response:

We thank the editor for pointing out the important issue. We have run the residual diagnostics and subsequently the ordinal mixed models for ratings of sincerity, happiness and anger. We have reported the results of the residual diagnostics in the main text as follows. We have also added S3 Table to include the results for the ordinal mixed models. The discussion regarding the floor effect observed for the ratings of anger was included in the limitation section.

(Page 14, Line 302) in Methods

According to the results of the residual diagnostics for all three ratings, we observed ceiling effects for the ratings of sincerity (p = .003) and happiness (p = .017) as well as a floor effect for the rating of anger (p < .001). To account for the skewness of the data, we also fitted the ordinal mixed models for each of the ratings, with condition and stimulus’ culture as predictors and with the same random effects as in LMMs.

(Page 18, Line 384) in Results

The results of ordinal mixed models (see S3 Table) for all three ratings were also consistent with those from LMMs.

(Page 30, Line 656) in Discussion

The floor effect of anger also implies the possibility that the participants did not consider the posers with smiles to have an underlying anger.

(Page 37, Line 798) in Supporting information

S3 Table. Results of Ordinal Mixed Models of the Effects of Condition and Stimulus’ Culture on the Evaluations of Sincerity, Happiness and Anger. The ordinal mixed models for the evaluations of the final smiles were conducted as a supplementary robustness check for the LMM results. The dependent variables included evaluations of sincerity, happiness and anger, respectively. The predictors for each of the evaluation included condition (neutral-to-smile condition and anger-to-smile condition) and stimulus’ culture (Japanese and Chinese). OR stands for odds ratio. CI stands for confidence interval.

Comment #5:

More time looking at the mouth generally means less time available for eyes/nose, and total looking time differs across participants and trials. You log-transform and z-score fixation durations, but the manuscript does not explain how zero fixations were handled, which is crucial for log transformation. You should clarify whether you used log(x), log(x+1), or another transformation. You should also consider using fixation proportions, total face-looking time as a covariate, or compositional approaches, because interpreting mouth fixation as an independent predictor can otherwise be misleading.

Response:

Thank you for pointing out the lack of explanation on the details regarding log transformation. We have used log(x+1) transformation for fixation duration and modified the main text in the following way specifying this detail.

We have also conducted additional analyses by adding total fixation duration to the face as covariate. There were significant main effects of fixation duration to the mouth for all three ratings as well as significant interactions wi

Attachment Submitted filename: Response to Reviewers.docx

# Peer review: Decision Letter 1

**DOI:** 10.1371/journal.pone.0356283.r003

**Authors:** Domna Banakou

2 Aug 2026

<p>Gaze allocation and evaluations of dynamic smiles following angry versus neutral expressions

PONE-D-26-07636R1

Dear Dr. Wu,

We’re pleased to inform you that your manuscript has been judged scientifically suitable for publication and will be formally accepted for publication once it meets all outstanding technical requirements.

Within one week, you’ll receive an e-mail detailing the required amendments. When these have been addressed, you’ll receive a formal acceptance letter and your manuscript will be scheduled for publication.

An invoice will be generated when your article is formally accepted. Please note, if your institution has a publishing partnership with PLOS and your article meets the relevant criteria, all or part of your publication costs will be covered. Please make sure your user information is up-to-date by logging into Editorial Manager at Editorial Manager® and clicking the ‘Update My Information' link at the top of the page. For questions related to billing, please contact billing support.

If your institution or institutions have a press office, please notify them about your upcoming paper to help maximize its impact. If they’ll be preparing press materials, please inform our press team as soon as possible -- no later than 48 hours after receiving the formal acceptance. Your manuscript will remain under strict press embargo until 2 pm Eastern Time on the date of publication. For more information, please contact onepress@plos.org.

Kind regards,

Domna Banakou

Academic Editor

PLOS One

Additional Editor Comments (optional):

Reviewers' comments:

Reviewer's Responses to Questions

**Comments to the Author**

Reviewer #1: All comments have been addressed

**********

2. Is the manuscript technically sound, and do the data support the conclusions?

Reviewer #1: (No Response)

**********

3. Has the statistical analysis been performed appropriately and rigorously?

Reviewer #1: (No Response)

**********

4. Have the authors made all data underlying the findings in their manuscript fully available?

The PLOS Data policy

Reviewer #1: (No Response)

**********

5. Is the manuscript presented in an intelligible fashion and written in standard English?

Reviewer #1: (No Response)

**********

Reviewer #1: (No Response)

**********

what does this mean?). If published, this will include your full peer review and any attached files.

If you choose “no”, your identity will remain anonymous but your review may still be made public.

**Do you want your identity to be public for this peer review?**  For information about this choice, including consent withdrawal, please see our Privacy Policy

Reviewer #1: **Yes:** Özge Karakale

**********

# Editor report: Acceptance letter

**DOI:** 10.1371/journal.pone.0356283.r004

**Authors:** Domna Banakou

PONE-D-26-07636R1

PLOS One

Dear Dr. Wu,

I'm pleased to inform you that your manuscript has been deemed suitable for publication in PLOS One. Congratulations! Your manuscript is now being handed over to our production team.

At this stage, our production department will prepare your paper for publication. This includes ensuring the following:

* All references, tables, and figures are properly cited

* All relevant supporting information is included in the manuscript submission,

* There are no issues that prevent the paper from being properly typeset

You will receive further instructions from the production team, including instructions on how to review your proof when it is ready. Please keep in mind that we are working through a large volume of accepted articles, so please give us a few days to review your paper and let you know the next and final steps.

Lastly, if your institution or institutions have a press office, please let them know about your upcoming paper now to help maximize its impact. If they'll be preparing press materials, please inform our press team within the next 48 hours. Your manuscript will remain under strict press embargo until 2 pm Eastern Time on the date of publication. For more information, please contact onepress@plos.org.

You will receive an invoice from PLOS for your publication fee after your manuscript has reached the completed accept phase. If you receive an email requesting payment before acceptance or for any other service, this may be a phishing scheme. Learn how to identify phishing emails and protect your accounts at https://explore.plos.org/phishing.

If we can help with anything else, please email us at customercare@plos.org.

Thank you for submitting your work to PLOS ONE and supporting open access.

Kind regards,

PLOS ONE Editorial Office Staff

on behalf of

Dr. Domna Banakou

Academic Editor

PLOS One
